# Targeting BRIX1 via Engineered Exosomes Induces Nucleolar Stress to Suppress Cancer Progression

**DOI:** 10.1002/advs.202407370

**Published:** 2024-10-30

**Authors:** Yu Gan, Qian Hao, Tao Han, Jing Tong, Qingya Yan, Hongguang Zhong, Bo Gao, Yanan Li, Zhisheng Xuan, Pengfei Li, Litong Yao, Yingying Xu, Yi‐Zhou Jiang, Zhi‐Ming Shao, Jun Deng, Jiaxiang Chen, Xiang Zhou

**Affiliations:** ^1^ Fudan University Shanghai Cancer Center and Institutes of Biomedical Sciences, Fudan University Shanghai 200032 P. R. China; ^2^ Department of Oncology, Shanghai Medical College Fudan University Shanghai 200032 P. R. China; ^3^ Institutes of Health Central Plains, Xinxiang Key laboratory for Molecular Oncology Xinxiang Medical University Xinxiang Henan 453003 P. R. China; ^4^ Department of Oncology The First Affiliated Hospital, Jiangxi Medical College, Nanchang University Nanchang Jiangxi 330006 P. R. China; ^5^ Jiangxi Key Laboratory for Individual Cancer Therapy Nanchang Jiangxi 330006 P. R. China; ^6^ Umibio Co. Ltd. Shanghai 201210 P. R. China; ^7^ Laboratory of Animal Center, Medical Experiment Center Shaanxi University of Chinese Medicine Xianyang 712046 P. R. China; ^8^ Department of Breast Surgery The First Hospital of China Medical University Shenyang Liaoning 110001 P. R. China; ^9^ Key Laboratory of Breast Cancer in Shanghai, Department of Breast Surgery Fudan University Shanghai Cancer Center, Fudan University Shanghai 200032 P. R. China; ^10^ Department of Physiology, School of Basic Medical Sciences Jiangxi Medical College, Nanchang University Nanchang 330006 P. R. China; ^11^ Shanghai Key Laboratory of Medical Epigenetics, International Co‐laboratory of Medical Epigenetics and Metabolism (Ministry of Science and Technology) Institutes of Biomedical Sciences, Fudan University Shanghai 200032 P. R. China

**Keywords:** BRIX1, engineered exosomes, nucleolar stress, p53, ribosome biogenesis, targeted therapy

## Abstract

Elevated ribosome biogenesis correlates with the rapid growth and progression of cancer. Targeted blockade of ribosome biogenesis induces nucleolar stress, which preferentially leads to the elimination of malignant cells. In this study, it is reported that the nucleolar protein BRIX1 is a critical regulator for the homeostasis between ribosome biogenesis and p53 activation. BRIX1 facilitated the processing of pre‐rRNA by supporting the formation of the PeBoW complex. In addition, BRIX1 prevented p53 activation in response to nucleolar stress by impairing the interactions between MDM2 and the ribosomal proteins, RPL5, and RPL11, thereby triggering the resistance of cancer cells to chemotherapy. Conversely, depletion of BRIX1 induced nucleolar stress, which in turn activated p53 through RPL5 and RPL11, consequently inhibiting the growth of tumors. Moreover, engineered exosomes are developed, which are surface‐decorated with iRGD, a tumor‐homing peptide, and loaded with siRNAs specific to BRIX1, for the treatment of cancer. iRGD‐Exo‐siBRIX1 significantly suppressed the growth of colorectal cancer and enhanced the efficacy of 5‐FU chemotherapy in vivo. Overall, the study uncovers that BRIX1 functions as an oncoprotein to promote rRNA synthesis and dampen p53 activity, and also implies that targeted inhibition of BRIX1 via engineered exosomes can be a potent approach for cancer therapy.

## Graphical abstract

Rapid growth and proliferation of cancer cells critically depend on robust ribosome biogenesis. BRIX1 plays an essential role in facilitating ribosomal RNA processing. Targeted inhibition of BRIX1 impairs ribosome biogenesis, triggers nucleolar stress, and activates the tumor suppressor p53. A strategy employing exosomes engineered to target BRIX1 is demonstrated to effectively suppress colorectal cancer both in vitro and in vivo.

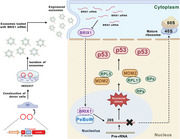

## 1 Introduction

Ribosome biogenesis is a multi‐step process that involves three fundamental steps: coordinated expression of ribosomal RNA (rRNA) and ribosomal proteins (RPs), the processing of rRNA precursors, and the assembly of 40S and 60S ribosome subunits in the nucleolus.^[^ ^1^, ^2^, ^3^ ^]^ Aberrant increases in the size and quantity of nucleoli, which is considered a significant marker for malignant cells, were linked to a poor prognosis of patients with cancer.^[^ ^4^, ^5^ ^]^ This is because cancer cells need to produce ribosomes at a higher rate than normal cells to meet the demand for protein synthesis during their rapid growth and proliferation.^[^ ^6^ ^]^ Various oncogenic signals have been found to promote the growth of cancer by increasing the production of ribosomes. For instance, c‐Myc facilitates the synthesis of all three types of rRNAs and the essential factors that are involved in ribosome biogenesis.^[^ ^7^ ^]^ Therefore, it is anticipated that targeting the nucleolar function could be an effective approach for eradicating cancer cells, while minimizing the cytotoxic effect on normal cells.

It has been well‐documented that perturbation of any crucial step of ribosome biogenesis may lead to nucleolar stress, also known as ribosomal stress.^[^ ^8^, ^9^, ^10^ ^]^ Under this stress condition, a portion of RPs, particularly RPL5 and RPL11, is translocated from the nucleolus to the nucleoplasm, where they interact with MDM2 and inhibit its E3 ligase activity toward p53, consequently resulting in the activation of p53.^[^ ^11^, ^12^, ^13^, ^14^ ^]^ In addition, nucleolar stress may lead to tumor suppression through p53‐independent signaling pathways. Several RPs, such as RPL5, RPL11, and RPS14, were shown to activate TAp73, a p53 homologue,^[^ ^15^ ^]^ and inactivate the oncoprotein c‐Myc^[^ ^16^, ^17^, ^18^, ^19^ ^]^ in response to nucleolar stress. Multiple strategies have been shown to trigger nucleolar stress. For example, a variety of chemotherapeutic agents can induce ribosome DNA (rDNA) damage or impede precursor rRNA (pre‐rRNA) processing, thus impairing ribosome biogenesis.^[^ ^20^, ^21^, ^22^ ^]^ Several compounds were developed to restrict rRNA synthesis by inhibiting the activity of RNA Polymerase (Pol) I, such as CX‐5461 and BMH‐21.^[^ ^23^, ^24^, ^25^ ^]^ However, it was later found that these agents also elicit DNA damage^[^ ^26^, ^27^ ^]^ or chromatin damage.^[^ ^28^ ^]^ Notedly, depleting certain RPs or nucleolar proteins that are essential for pre‐rRNA processing was demonstrated to specifically induce nucleolar stress in laboratory settings.^[^ ^29^, ^30^, ^31^, ^32^ ^]^ These findings have motivated us to further investigate the potential of triggering nucleolar stress as a therapeutic strategy for cancer treatment.

Using a combination of bioinformatics analysis and cell‐based assays, we identified the nucleolar protein BRIX1 as a key regulator of ribosome biogenesis and nucleolar stress. The Brix (biogenesis of ribosomes in Xenopus) was first detected in frog and later found to participate in rRNA processing in yeast.^[^ ^33^, ^34^ ^]^ The expression of human BRIX1 was recently shown to correlate with rRNA synthesis and promote the translation of GLUT1 in colorectal cancer.^[^ ^35^ ^]^ However, the role and mechanism of BRIX1 in rRNA synthesis and cancer development remain largely elusive. In this study, we found that BRIX1 mainly facilitated the processing of 12S and 32S rRNA precursors by binding to the PES1‐BOP1‐WDR12 (PeBoW) complex. Depletion of BRIX1 elicited nucleolar stress to activate p53, whereas overexpression of BRIX1 alleviated nucleolar stress‐induced p53 activation by preventing the interactions between RPL5/RPL11 and MDM2. Our results also showed that BRIX1 was highly expressed in breast and colorectal cancers and higher levels of BRIX1 correlated with worse prognoses. These findings suggested that BRIX1 might be a feasible target for cancer treatment. Remarkably, we developed a delivery system utilizing engineered exosomes that encapsulated small interfering RNAs (siRNAs) against BRIX1. This Exo‐siBRIX1 was demonstrated to efficiently suppress the growth of cancer in vivo. Altogether, our study uncovers a crucial role for BRIX1 in regulating the nucleolar stress‐p53 pathway and provides a potential BRIX1‐targeting strategy for the treatment of cancer.

## 2 Results

### 2.1 Identification of the Nucleolar Protein BRIX1 as a Regulator for p53

**Figure 1:**
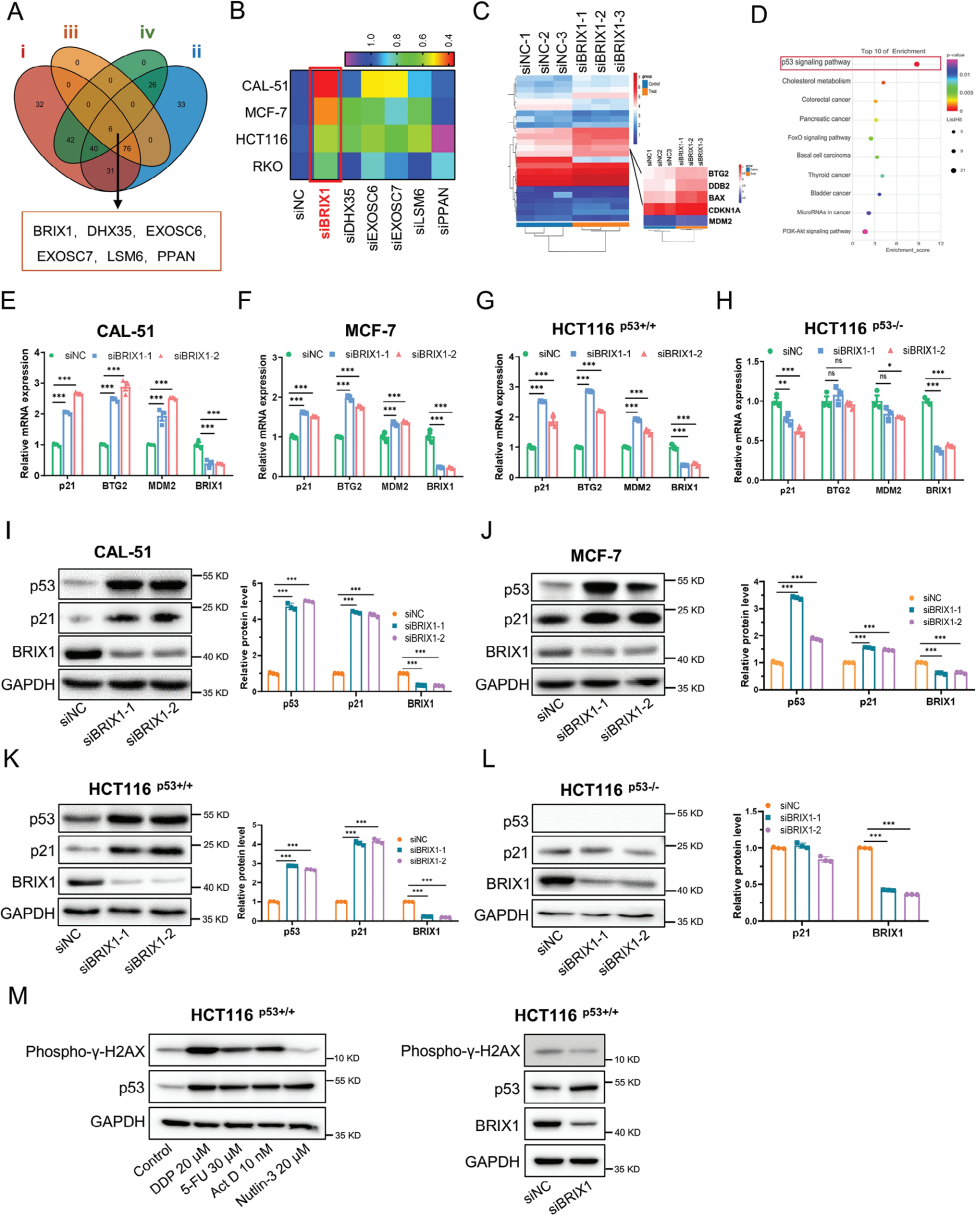
Ablation of BRIX1 induces p53 activation. A) The Venn diagram analysis depicts the screening for potential regulators of ribosome biogenesis. B) The heatmap shows that BRIX1 depletion dramatically inhibits cell growth in four cancer cell lines through cell viability assays. C) The heatmap displays that the expression of numerous p53 target genes is elevated in BRIX1‐depleted CAL‐51 cells through an RNA‐sequencing analysis. D) The Kyoto Encyclopedia of Genes and Genomes (KEGG) analysis shows that genes in the p53 pathway are enriched in BRIX1‐depleted cells. E‐G) Knockdown of BRIX1 elevates mRNA levels of p53 target genes. CAL‐51 (E), MCF‐7 (F), and HCT116^p53+/+^ (G) cells were transfected with control or BRIX1 siRNAs for 48 h, followed by RT‐qPCR analysis. H) Knockdown of BRIX1 has no impact on mRNA levels of p53 target genes in HCT116 ^p53−/−^ cells. I‐K) Knockdown of BRIX1 elevates protein levels of p53 and its target genes. CAL‐51 (I), MCF‐7 (J), and HCT116^p53+/+^ (K) cells were transfected with control or BRIX1 siRNAs for 48 h, followed by IB analysis. L) Knockdown of BRIX1 has no impact on protein levels of p53 target genes in HCT116 ^p53−/−^ cells. M) Knockdown of BRIX1 does not increase γ‐H2AX phosphorylation. Cells were treated with agents for 24 h or transfected with siRNAs for 48 h, followed by IB analysis. ***p < 0.001.

Because of the essential roles of ribosome homeostasis in harnessing p53 activity and supporting cancer development, we investigated the nucleolar proteins that might be critical for ribosome biogenesis. It has been identified that 286 nucleolar proteins are involved in pre‐rRNA processing in yeast.^[^ ^36^ ^]^ These nucleolar proteins were selected based on the following criteria: (i) proteins that directly control pre‐rRNA processing in yeast; (ii) proteins with human counterparts; (iii) proteins that possibly have a comparable function in ribosome biogenesis in both human and yeast; and (iv) proteins whose role in cancer is not well understood. Thus, we obtained six nucleolar proteins: BRIX1, DHX35, EXOSC6, EXOSC7, LSM6, and PPAN for further analysis (**Figure** **1**A). To test if these nucleolar proteins are essential for the growth of cancer cells, we performed a cell viability assay by knocking down each of the six nucleolar proteins in four different cancer cell lines, including CAL‐51, MCF‐7, HCT116, and RKO. Our results revealed that depletion of BRIX1 inhibited the growth of all cell lines more dramatically compared to knockdown of the other nucleolar proteins (Figure 1B). To explore the potential signaling pathways that are regulated by BRIX1, we performed an RNA‐sequencing (RNA‐seq) analysis by depleting BRIX1 in CAL‐51 breast cancer cells. The heatmap and Kyoto Encyclopedia of Genes and Genomes (KEGG) analysis displayed that ablation of BRIX1 activated the p53 pathway (Figure 1C,D). To validate this result, we knocked down BRIX1 expression by two independent siRNAs and found that BRIX1 deficiency remarkably elevated the mRNA expression of p53 target genes, such as CDKN1A (also known as p21), BTG2, and MDM2 in CAL‐51, MCF‐7, and HCT116 cells (Figure 1E–G). Also, we observed that knockdown of any of the other five nucleolar proteins induced p21 expression, while BRIX1 depletion exhibited a more marked effect on p21 induction in HCT116 ^p53+/+^ cells (Figure S1A, Supporting Information). Consistently, knockdown of BRIX1 also induced the expression of p53 and p21 at the protein level (Figure 1I–K). The upregulation of p21, BTG2, and MDM2 was dependent on p53, as this was not observed in HCT116 ^p53−/−^ cells (Figure 1H,L). The level of p21 was even reduced upon BRIX1 knockdown, possibly through a p53‐independent regulatory mechanism of p21 expression.^[^ ^37^ ^]^ In addition, our results showed that BRIX1 depletion did not significantly affect the mRNA expression of p53 (Figure S1B, Supporting Information) or the levels of mutant p53 (Figure S1C,D, Supporting Information). Interestingly, in contrast to the effect of cisplatin (DDP), 5‐fluorouracil (5‐FU), or actinomycin D (Act D) treatment, knockdown of BRIX1 did not lead to an increase in γ‐H2AX phosphorylation (Figure 1M), indicating that there was no DNA damage stress induced. Together, these results suggest that the nucleolar protein BRIX1 may act as a regulator for ribosome biogenesis and the p53 pathway, which is detailed below.

### 2.2 BRIX1 Facilitates the Processing of Pre‐rRNA via the PeBoW Complex

**Figure 2:**
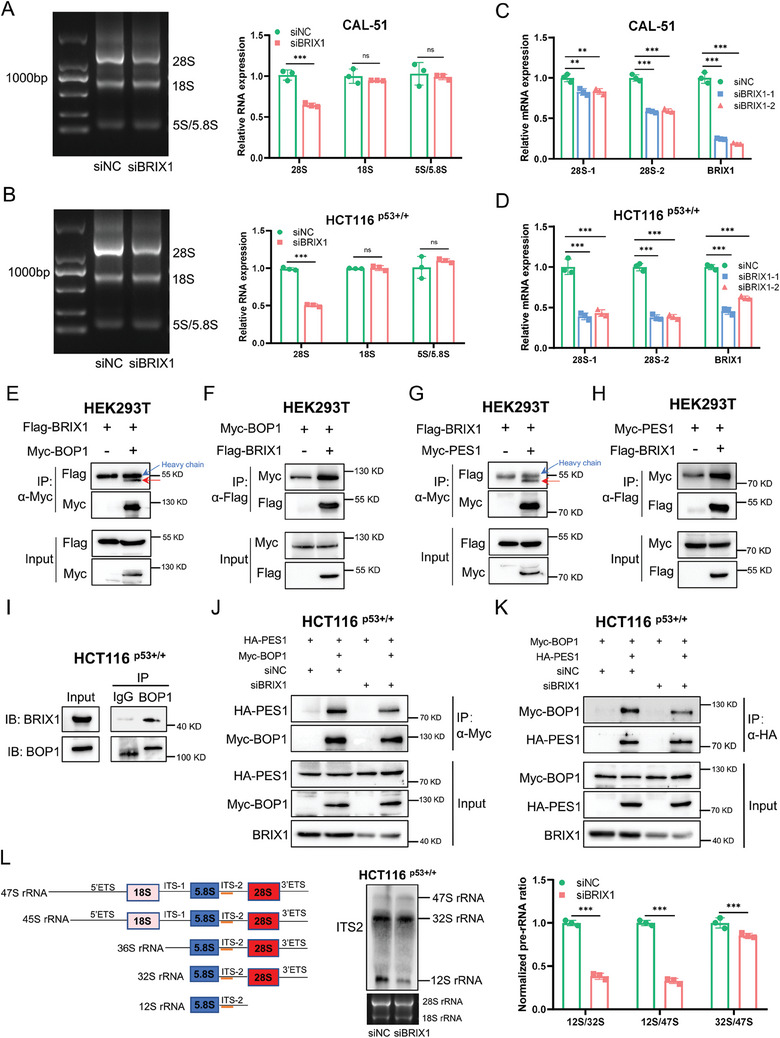
BRIX1 promotes pre‐rRNA processing through the PeBoW complex. A, B) Knockdown of BRIX1 reduces levels of 28S rRNA. CAL‐51 (A) and HCT116 p53^+/+^ (B) cells were transfected with control or BRIX1 siRNA for 48 h, followed by agarose gel electrophoresis and quantification with ImageJ. C, D) Knockdown of BRIX1 reduces levels of 28S rRNA determined by RT‐qPCR. E‐H) BRIX1 interacts with BOP1 and PES1. Cells were transfected with the indicated plasmids for 48 h, followed by co‐IP‐IB analysis using antibodies as indicated. The blue arrows indicate the bands for the heavy chain, while the red arrows denote the Flag‐tagged protein in E and G. I) Endogenous interaction of BRIX1 with BOP1. HCT116 ^p53+/+^ cells were treated with MG132 for 6 h and subjected to co‐IP‐IB analysis. J, K) Knockdown of BRIX1 impedes the interaction of BOP1 with PES1. Cells were transfected with the indicated siRNAs and plasmids, followed by co‐IP‐IB analysis using antibodies as indicated. L) Knockdown of BRIX1 impairs pre‐rRNA processing. Cells were transfected with control or BRIX1 siRNA, followed by Northern blotting analysis. A schematic illustration of pre‐rRNA processing and the probe against ITS‐2 (orange) used in the experiment is shown in the left panel. ***p < 0.001.

It has been reported that Brix is involved in the processing of pre‐rRNA and the synthesis of large subunits of ribosomes in yeast.^[^ ^33^, ^34^ ^]^ However, it remained unclear if BRIX1 has a conserved function in human cells. To clarify this, we performed a gel electrophoresis assay to analyze total RNAs in CAL‐51 and HCT116 ^p53+/+^ cells, and the results showed that the levels of 28S rRNA were significantly reduced when BRIX1 was depleted (**Figure** **2**A,B). These results were also confirmed by RT‐qPCR analysis in both cell lines (Figure 2C,D). To investigate the underlying mechanism, we utilized the Search Tool for the Retrieval of Interacting Genes/proteins (STRING) database to predict potential interacting proteins of BRIX1, revealing the identification of BOP1, PES1, and WDR12 (Figure S2A, Supporting Information). These three proteins constitute the PeBoW complex that is essential for the processing of pre‐rRNA and maturation of the 60S ribosomal subunit in mammalian cells.^[^ ^38^, ^39^ ^]^ Thus, we speculated that BRIX1 might promote rRNA synthesis by interacting with the PeBoW complex. To test this hypothesis, we performed a set of reciprocal co‐immunoprecipitation (co‐IP) assays and found that exogenous BRIX1 interacted with both exogenous BOP1 and PES1 (Figure 2E–H), but not with WDR12 (Figure S2B,C, Supporting Information). Because the level of endogenous BOP1 is essential for the formation of the PeBoW complex,^[^ ^40^ ^]^ we further tested whether BRIX1 bound to and regulated the level of endogenous BOP1. While the endogenous interaction between BRIX1 and BOP1 was observed in cancer cells (Figure 2I), the protein levels of BOP1 were not affected by knocking down BRIX1 (Figure S2D, Supporting Information). Remarkably, our results revealed that depletion of BRIX1 drastically decreased the interaction between BOP1 and PES1, as evidenced by reciprocal co‐IP assays (Figure 2J,K), suggesting that BRIX1 is required for the formation of the PeBoW complex. As the PeBoW complex contributes to the synthesis and processing of 32S pre‐rRNA,^[^ ^41^ ^]^ we performed a Northern blot analysis using a digoxin‐labeled internal transcribed spacer 2 (ITS2) probe that could specifically detect 47S, 32S, and 12S pre‐rRNAs. Our result showed that depletion of BRIX1 mainly impaired the processing of 32S pre‐rRNA, resulting in a marked reduction of 12S pre‐rRNA, while the processing of 47S pre‐rRNA might be also slightly affected (Figure 2L). Together, these results demonstrate that BRIX1 is required for pre‐rRNA processing by regulating the stability of the PeBoW complex.

### 2.3 BRIX1 Deficiency Inhibits the Growth of Cancer Cells by Activating the Nucleolar Stress‐p53 Pathway

**Figure 3:**
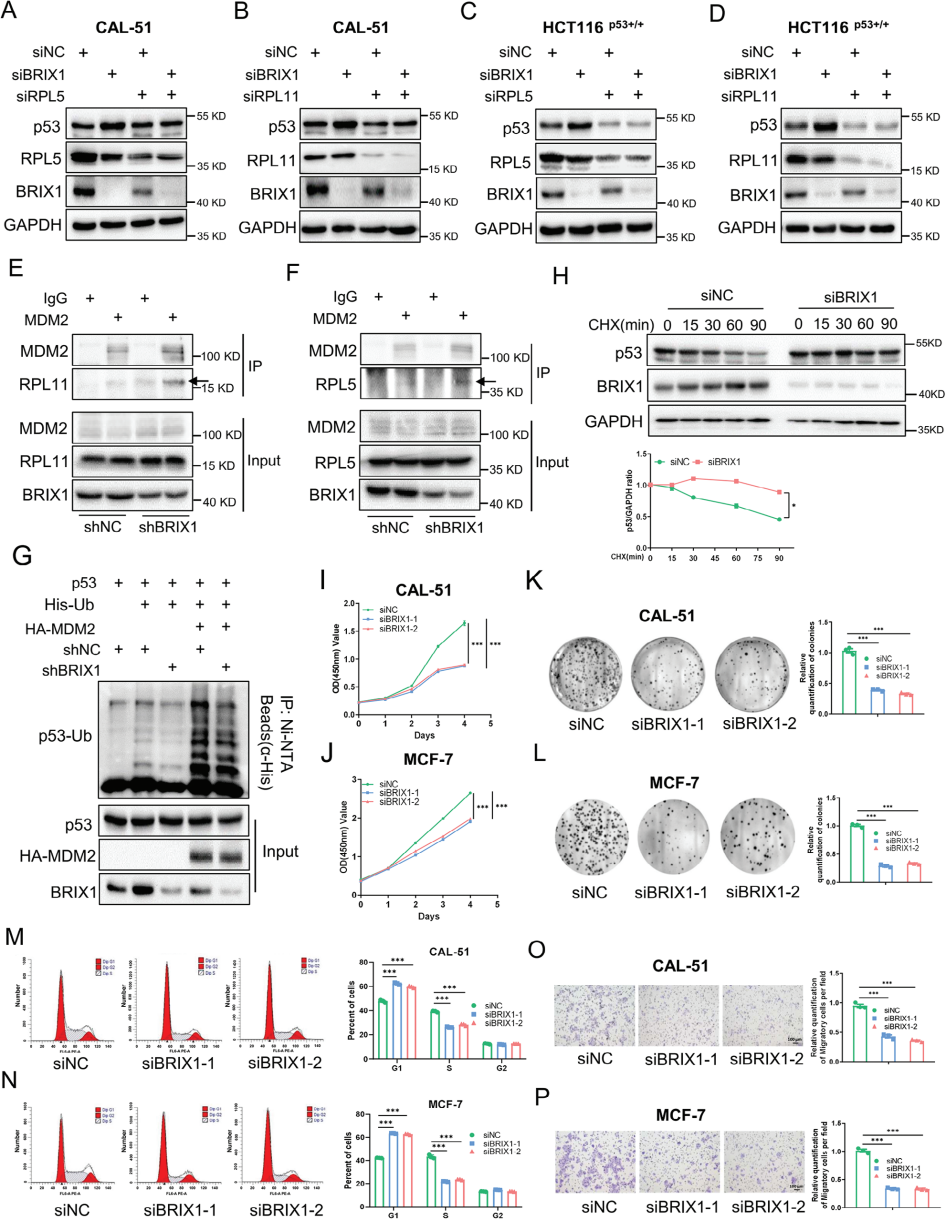
Ablation of BRIX1 activates the nucleolar stress‐p53 pathway to suppress the growth of cancer. A‐D) Knockdown of RPL5 or RPL11 impairs the activation of p53 upon BRIX1 depletion. CAL‐51 (A, B) and HCT116 ^p53+/+^ C, D) cells were transfected with siRNAs as indicated for 48 h, followed by IB analysis. E, F) Knockdown of BRIX1 increases the interactions of MDM2 with RPL5 and RPL11, respectively. Cells stably expressing shNC or shBRIX1 were used for co‐IP‐IB analysis with antibodies as indicated. The proteasome inhibitor MG132 was supplemented into the medium for 5 h before cell harvest. G) Knockdown of BRIX1 diminishes p53 ubiquitination induced by MDM2. HCT116 ^p53−/−^ cells stably expressing control or BRIX1 shRNA were transfected with plasmids as indicated for 48 h and treated with MG132 for 5 h, followed by in vivo ubiquitination assay and IB analysis. H) Knockdown of BRIX1 extends the half‐life of p53 protein. Cells were transfected with control or BRIX1 siRNA. Cycloheximide (CHX) (100 mg mL^−1^) was supplemented into the medium for the indicated time before cells were harvested for IB analysis. I, J) Knockdown of BRIX1 inhibits the proliferation of breast cancer cells with wild‐type p53. CAL‐51 (I) and MCF‐7 (J) cells were transfected with control or BRIX1 siRNAs for 6–12 h and seeded in 96‐well plates for a cell viability assay. K, L) Knockdown of BRIX1 impedes the colony‐forming ability of wild‐type p53‐harboring cancer cells. CAL‐51 (K) and MCF‐7 (L) cells were transfected with control or BRIX1 siRNAs for 6–12 h and were seeded in 6‐well plates for about 14 days. Colonies were fixed with methanol, and visualized by crystal violet staining. M, N) Knockdown of BRIX1 induces G1‐cell cycle arrest in breast cancer cells harboring wild‐type p53. CAL‐51 (M) and MCF‐7 (N) cells were transfected with control or BRIX1 siRNAs for 48 h, followed by flow cytometric analysis. O, P) Knockdown of BRIX1 represses the migration of wild‐type p53‐harboring cancer cells. CAL‐51 (O) and MCF‐7 (P) cells were transfected with control or BRIX1 siRNAs for 6–12 h, followed by a cell migration assay. ***p < 0.001.

Since BRIX1 is required for the processing of 32S pre‐rRNA, we tested if BRIX1 ablation could trigger nucleolar stress by performing immunofluorescence (IF) staining of the nucleolar marker, NPM1. As expected, knockdown of BRIX1 led to the relocation of NPM1 from the nucleolus to the nucleoplasm (Figure S2E, Supporting Information), indicating that BRIX1 deficiency induced nucleolar stress. It is known that the activation of p53 upon nucleolar stress requires the interactions of RPL5/RPL11 with MDM2 and the consequent inhibition of MDM2.^[^ ^29^, ^30^, ^31^, ^32^, ^42^, ^43^ ^]^ This is also evidenced by the fact that cancer cells expressing an MDM2 mutant that cannot bind to RPL5 and RPL11 display resistance to p53 activation induced by nucleolar stress.^[^ ^14^ ^]^ We then elucidated whether knockdown of BRIX1 activated the p53 pathway through RPL5 and RPL11. Our results showed that depletion of RPL5 or RPL11 completely abolished the activation of p53 caused by BRIX1 deficiency in CAL‐51 cells and HCT116 ^p53+/+^ cells (**Figure** **3**A–D). We noticed that knockdown of BRIX1 moderately reduced the levels of RPL5 and RPL11, possibly due to the fact that ribosomal components could mutually stabilize each other as previously described.^[^ ^30^, ^44^ ^]^ In addition, we tested whether BRIX1 deficiency enhanced the interaction of MDM2 with RPL5 or RPL11 by co‐IP assays. MDM2 could slightly bind to RPL5 and RPL11 under normal growing conditions, while knockdown of BRIX1 markedly increased the binding of MDM2 to these two RPs (Figure 3E,F). The increased interactions between MDM2 and RPs may impair the E3 ligase activity of MDM2 toward p53.^[^ ^8^, ^9^, ^10^ ^]^ Thus, we performed an in vivo ubiquitination assay to test if BRIX1 deficiency had an impact on MDM2‐mediated ubiquitination of p53. The results showed that overexpression of MDM2 promoted the ubiquitination of p53, whereas knockdown of BRIX1 reduced p53 ubiquitination caused by MDM2 (Figure 3G). Consistently, depletion of BRIX1 significantly extended the half‐life of p53 protein, as shown in cycloheximide‐chase analysis (Figure 3H). These results demonstrate that BRIX1 deficiency triggers nucleolar stress, which leads to increased interactions between RPL5/RPL11 and MDM2, ultimately resulting in the activation of p53.

Next, we investigated the role of BRIX1 in wild‐type p53‐harboring cancer cells by knocking down its expression. Our results showed that ablation of BRIX1 significantly suppressed the proliferation (Figure 3I,J) and colony formation (Figure 3K,L) of CAL‐51 and MCF‐7 cells. Consistently, ablation of BRIX1 induced G1‐cell cycle arrest (Figure 3M,N) and apoptosis (Figure S2F,G, Supporting Information). Moreover, the migration of cancer cells was also dramatically inhibited by depleting BRIX1 (Figure 3O,P). Taken together, our findings demonstrate that depletion of BRIX1 activates the nucleolar stress‐p53 pathway to suppress the growth and propagation of cancer cells.

### 2.4 Excessive BRIX1 Prevents Nucleolar Stress‐Induced Activation of p53

**Figure 4:**
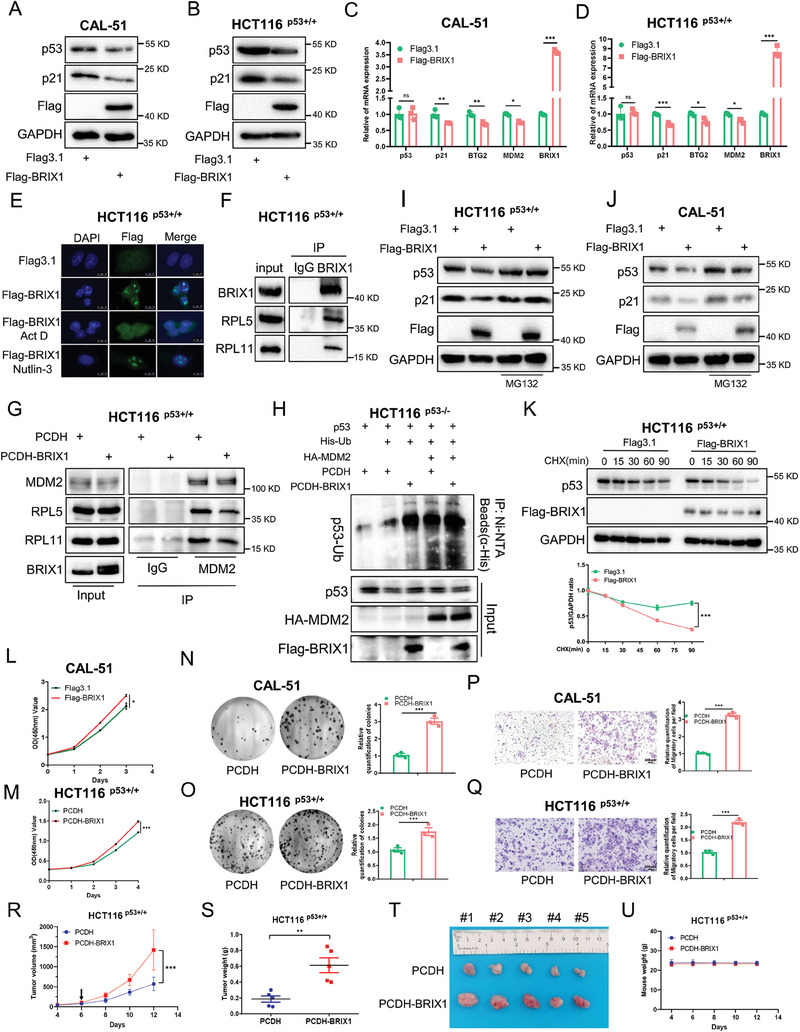
Overexpression of BRIX1 impairs p53 activation in response to nucleolar stress. A‐D) Overexpression of BRIX1 inhibits the expression of p53 and its target genes upon nucleolar stress. CAL‐51 (A, C) and HCT116 ^p53+/+^ B, D) cells were transfected with plasmids as indicated and treated with Act D (10 nM) for 24 h, followed by IB or RT‐qPCR analysis. E) BRIX1 translocates from the nucleolus to the nucleoplasm in cells when treated with Actinomycin D (Act D) (10 nM), but not with Nutlin‐3 (20 µM). Cells were transfected with plasmids and treated with agents as indicated, followed by IF staining. F) BRIX1 interacts with RPL5 and RPL11. HCT116 ^p53+/+^ cells were treated with Act D (10 nM) for 24 h and MG132 (20 µM) for 6 h, followed by co‐IP‐IB analysis. G) BRIX1 prevents the interactions between MDM2 and RPL5/RPL11. HCT116 ^p53+/+^ cells were transfected with plasmids as indicated and treated with Act D (10 nM) for 24 h and MG132 (20 µM) for 6 h, followed by co‐IP‐IB analysis. H) Overexpression of BRIX1 increases the ubiquitination of p53. HCT116 ^p53−/−^ cells stably overexpressing control or BRIX1 were transfected with the indicated plasmids for 48 h, and treated with Act D (10 nM) for 24 h and MG132 (20 µM) for 6 h, followed by in vivo ubiquitination assay. I, J) HCT116 ^p53+/+^ and CAL‐51 cells were transfected with plasmids as indicated and treated with Act D (10 nM) for 24 h and MG132 (20 µM) for 6 h, followed by IB analysis. K) Overexpression of BRIX1 shortens the half‐life of p53 protein. HCT116 ^p53+/+^ cells were transfected with plasmids as indicated and treated with Act D (10 nM) for 24 h and CHX (100 mg/ml) at different time points, followed by IB analysis. L, M) Overexpression of BRIX1 increases the proliferation of CAL51 (L) and HCT116 ^p53+/+^ (M) cells. Cells were transfected with plasmids as indicated and treated with Act D (10 nM), followed by a cell viability assay. N, O) Overexpression of BRIX1 enhances the colony‐forming ability of CAL51 (N) and HCT116 ^p53+/+^ (O) cells. Cells stably overexpressing control or BRIX1 were treated with Act D (10 nM), followed by a colony formation assay. P, Q) Overexpression of BRIX1 increases the migration of CAL51 (P) and HCT116 ^p53+/+^ (Q) cells. Cells stably overexpressing control or BRIX1 were treated with Act D (10 nM), followed by a cell migration assay. *p < 0.05, **p < 0.01, ***p < 0.001. R‐U) Overexpression of BRIX1 promotes the growth rate (R), weight (S), and size (T) of HCT116 ^p53+/+^ cell‐derived xenograft tumors, while does not affect mouse body weight (U). 30 µg kg^−1^ Act D was administered intraperitoneally on the indicated day post‐inoculation. Data are presented as mean ± SD, *n* = 5. **p < 0.01, ***p < 0.001.

We then investigated the potential impact of excessive BRIX1 on p53 activity. To our surprise, the expression levels of p53 were not affected by overexpressing BRIX1 in cancer cells under normal growing conditions (Figure S3A,B, Supporting Information). Thus, we sought to examine if ectopic BRIX1 regulated p53 under cellular stress conditions. Interestingly, our results showed that ectopic BRIX1 dramatically reduced the expression of p53 protein (**Figure** **4**A,B) and its target genes (Figure 4C,D) in cancer cells when treated with a low dose of Act D, as well as upon the treatment with DDP or 5‐FU (Figure S3C,D, Supporting Information). However, ectopic BRIX1 failed to regulate the p53 level in cancer cells when treated with Nutlin‐3, an inducer of p53 by antagonizing MDM2 (Figure S3E, Supporting Information). It is known that Act D at a low concentration preferentially inhibits RNA Pol I activity,^[^ ^12^ ^]^ while both DDP and 5‐FU impair ribosome biogenesis possibly by inducing rDNA damage or inhibiting pre‐rRNA processing.^[^ ^20^, ^21^ ^]^ Therefore, these results suggested that BRIX1 repressed the activation of p53 caused by nucleolar stress.

To elucidate how BRIX1 inhibits the expression of p53, we first examined the subcellular localization of BRIX1. Our results showed that BRIX1 was mainly distributed in the nucleolus under non‐stress conditions or upon Nultin‐3 treatment, but it could be relocated from the nucleolus to the nucleoplasm in response to nucleolar stress caused by Act D (Figure 4E). We then wondered if ectopic BRIX1 prevented the interactions between RPs and MDM2 that occur in the nucleoplasm upon nucleolar stress. The co‐IP assays revealed that BRIX1 bound to both RPL5 and RPL11 in cancer cells when treated with Act D (Figure 4F). In addition, ectopic BRIX1 reduced the interactions of MDM2 with RPL5 and RPL11 (Figure 4G), possibly because BRIX1 competed with MDM2 for the binding to these two RPs. Furthermore, we found that ectopic BRIX1 enhanced MDM2‐induced ubiquitination of p53 (Figure 4H), while the degradation of p53 caused by BRIX1 overexpression could be blocked by the proteasome inhibitor MG132 (Figure 4I,J). Finally, the cycloheximide‐chase analysis showed that BRIX1 overexpression shortened the half‐life of p53 protein (Figure 4K). These results demonstrate that BRIX1 translocates into the nucleoplasm to prevent the interactions of MDM2 with RPL5/RPL11, thereby inhibiting nucleolar stress‐induced stabilization and activation of p53.

Next, we tested whether ectopic BRIX1 promoted the survival and growth of cancer cells under nucleolar stress. Our results revealed that overexpression of BRIX1 significantly increased the proliferation (Figure 4L,M) and colony formation (Figure 4N,O), while reduced apoptosis (Figure S3F,G, Supporting Information) of cancer cells when treated with a low dose of Act D. In addition, ectopic BRIX1 promoted the migration of cancer cells (Figure 4P,Q). On the contrary, ectopic BRIX1 had no significant impact on the growth and colony formation of HCT116 ^p53−/−^ cells (Figure S3H,I, Supporting Information). Moreover, we determined the role of BRIX1 in vivo by establishing a xenograft tumor model. In line with the cell‐based results, stable overexpression of BRIX1 in HCT116 ^p53+/+^ cells significantly promoted the growth rate, weight, and size of xenograft tumors derived from these cells (Figure 4R–T) without affecting the average mouse body weight (Figure 4U). Together, these results demonstrate that excessive BRIX1 counteracts nucleolar stress‐mediated anti‐tumor effects by inactivating p53, suggesting the involvement of BRIX1 in fostering resistance to chemotherapy.

### 2.5 The Tumor‐Inhibitory Effects of Depleting BRIX1 are Largely Dependent on p53

**Figure 5:**
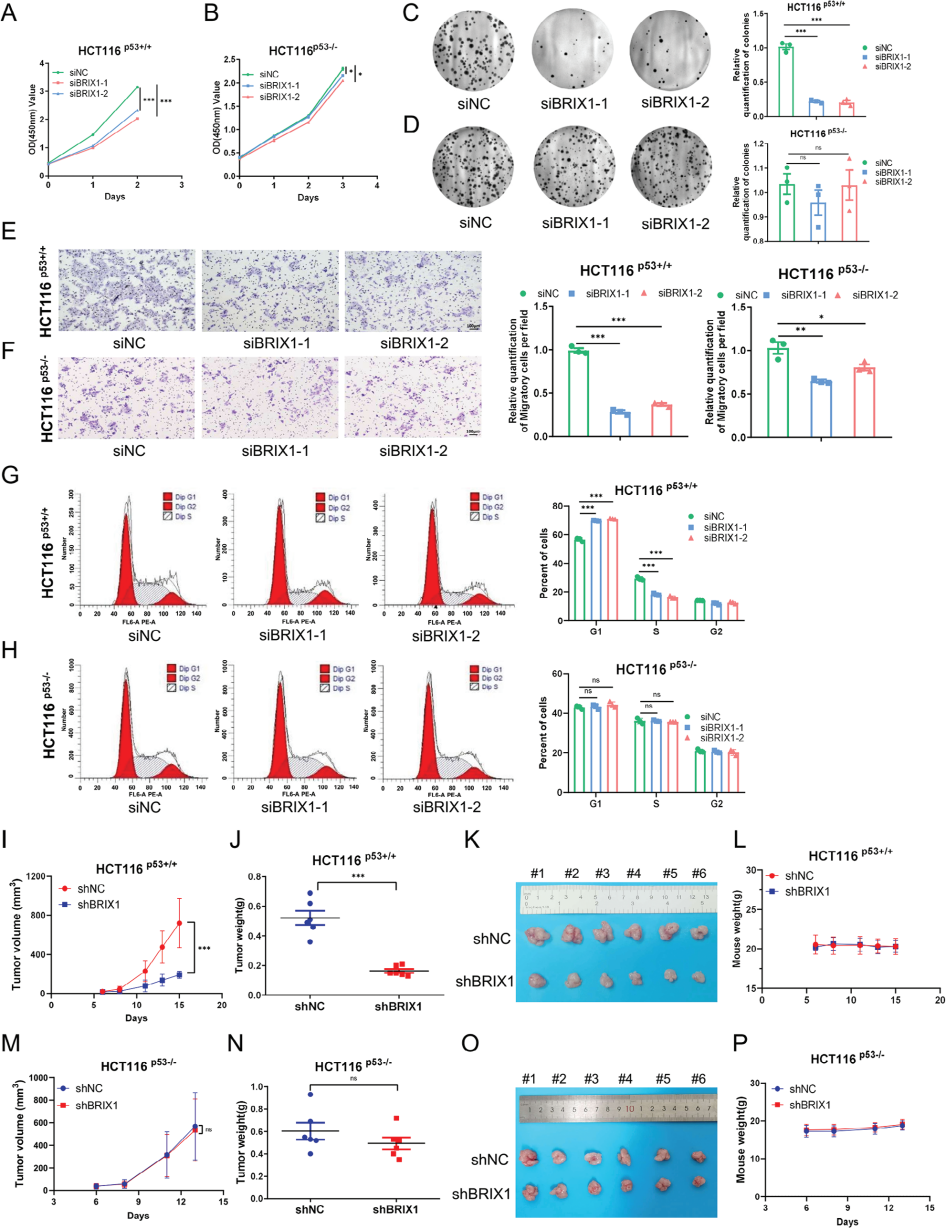
The tumor‐inhibitory effects of depleting BRIX1 are largely dependent on p53. A, B) Knockdown of BRIX1 suppresses the growth of HCT116 ^p53+/+^ cells (A) more dramatically than HCT116 ^p53−/−^ cells (B). Cells were transfected with siRNAs as indicated and subjected to a cell viability assay. C, D) Knockdown of BRIX1 reduces the colony‐forming ability of HCT116 ^p53+/+^ cells (C) more dramatically than HCT116 ^p53−/−^ cells (D). Cells were transfected with siRNAs as indicated and subjected to a colony formation assay. E, F) Knockdown of BRIX1 impedes the migration of HCT116 ^p53+/+^ cells (E) more dramatically than HCT116 ^p53−/−^ cells (F). Cells were transfected with siRNAs as indicated and subjected to a cell migration assay. (G, H) Knockdown of BRIX1 induces G1‐arrest in HCT116 ^p53+/+^ G) but not HCT116 ^p53−/−^ cells H). Cells were transfected with siRNAs as indicated, followed by flow cytometric analysis. I‐P) BRIX1 depletion dramatically suppresses the growth rate (I), weight (J), and size (K) of HCT116 ^p53+/+^ cell‐derived tumors, but has a marginal effect on HCT116 ^p53−/−^ cell‐derived tumors (M‐O). Mouse body weight is not affected (L, P). Data are presented as mean ± SD, *n* = 6. *p < 0.05, **p < 0.01, ***p < 0.001.

Since BRIX1 played an essential role in regulating p53 activity, we wondered whether or not BRIX1 depletion suppressed colorectal cancer in a p53‐dependent fashion. To this end, we employed the isogenic colorectal cancer cell lines, HCT116 ^p53+/+^ and HCT116 ^p53−/−^. Our results showed that knockdown of BRIX1 dramatically inhibited the proliferation of HCT116 ^p53+/+^ cells, while only slightly inhibited the proliferation of HCT116 ^p53−/−^ cells (**Figure** **5**A,B). Consistently, knockdown of BRIX1 had a more significant impact on the colony‐forming ability of HCT116 ^p53+/+^ cells compared to HCT116 ^p53−/−^ cells (Figure 5C,D). Moreover, the inhibitory effect of depleting BRIX1 on the migration of colorectal cancer cells was also partially dependent on p53 status (Figure 5E,F). These results suggested that BRIX1 depletion could moderately inhibit the growth and migration of cancer cells by perturbing ribosome biogenesis in the absence of p53. However, the induction of G1‐cell cycle arrest by depleting BRIX1 was exclusively dependent on the presence of p53 (Figure 5G,H), as this effect was mediated by the p53 target gene p21 (Figure 1E–L). In addition, knockdown of BRIX1 moderately suppressed the growth of SW480 and MDA‐MB‐231 cells with mutant p53 (Figure S1E,F, Supporting Information), possibly due to the impairment of rRNA processing. Finally, we tested whether depleting BRIX1 suppressed tumor growth through p53 in vivo. Our results revealed that BRIX1 depletion markedly reduced the growth rate, weight, and size of xenograft tumors derived from HCT116 ^p53+/+^ cells (Figure 5I–L), whereas had a marginal effect on the growth of xenograft tumors derived from HCT116 ^p53−/−^ cells (Figure 5M–P). These results demonstrate that depleting BRIX1 has a more significant effect on colorectal cancer cells with wild‐type p53 compared to those lacking p53.

### 2.6 High Levels of BRIX1 Correlate with Poor Prognoses in Breast and Colorectal Cancers

**Figure 6:**
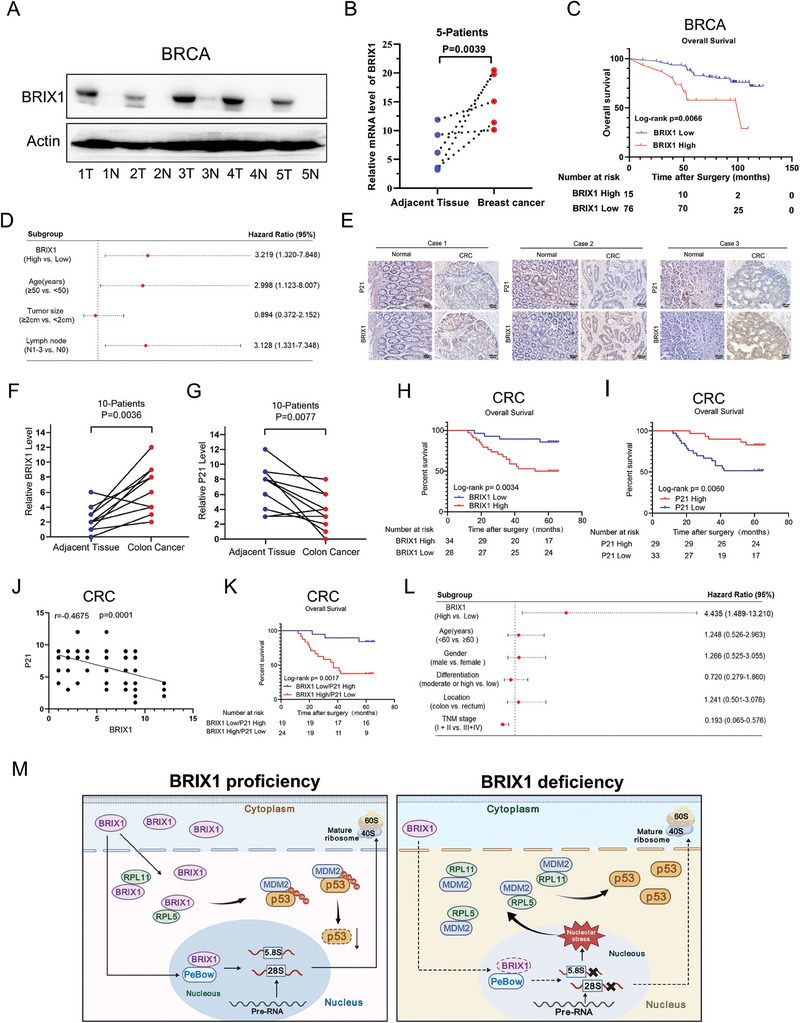
BRIX1 is highly expressed in breast and colorectal cancer tissues and is associated with unfavorable prognoses. A, B) The protein and mRNA levels of BRIX1 are elevated in breast cancer samples compared to adjacent normal tissues. Five pairs of fresh tissues were analyzed by IB and RT‐qPCR, respectively. C, D) Higher expression level of BRIX1 is a worse prognostic factor in breast cancer. E–G) Representative images E) and graphs of ten pairs of samples (F, G) show that the level of BRIX1 expression is higher while p21 expression is lower in colorectal cancer tissues when compared to paired adjacent normal tissues. H, I) Higher level of BRIX1 (H) or lower level of p21 (I) is associated with worse overall survival in a cohort of 62 colorectal cancer patients. J, K) High level of BRIX1 combined with low level of p21 is more significantly associated with worse overall survival. L) Higher expression level of BRIX1 is a worse prognostic factor in colorectal cancer. M) A schematic diagram for the role of BRIX1 in cancer progression and treatment. High level of BRIX1 facilitates pre‐rRNA processing and promotes MDM2‐mediated p53 degradation (left panel). Depleting BRIX1 activates the p53 pathway by inducing nucleolar stress (right panel).

Considering that BRIX1 was necessary for the survival and proliferation of breast and colorectal cancer cells, we investigated the clinical significance of BRIX1 in these two types of cancer. First, we assessed the expression of BRIX1 in breast cancer and matched normal tissues by IB and RT‐qPCR analyses. Our results showed that the protein and mRNA levels of BRIX1 were elevated in breast cancer tissues compared to normal tissues (**Figure** **6**A,B). In addition, immunohistochemistry (IHC) analysis of 91 breast cancer tissues revealed that higher expression of BRIX1 was significantly associated with higher tumor/node/metastasis (TNM) stages and worse overall survival of patients (Figure 6C; Table S1, Supporting Information). Moreover, univariate and multivariate Cox regression analysis further confirmed that high expression of BRIX1 was a poor prognostic factor in breast cancer (Figure 6D; Table S2, Supporting Information). Meanwhile, we determined the clinical relevance of BRIX1 levels using colorectal cancer samples with low expression of p53, as p53‐low samples might contain wild‐type p53.^[^ ^45^ ^]^ Our IHC analysis showed that BRIX1 was expressed at higher levels in colorectal cancer compared to normal tissues, whereas p21 was expressed at lower levels in colorectal cancer than normal tissues (Figure 6E–G). This finding was partially consistent with our cell‐based results (Figure 4A–D). By evaluating the expression of BRIX1 and p21 in 62 colorectal cancer samples, we found that elevated levels of BRIX1 were associated with advanced TNM stages and a poor prognosis (Figure 6H; Table S3, Supporting Information), while high levels of p21 correlated with a favorable prognosis (Figure 6I). Also, our analysis revealed an inverse correlation between the expression of BRIX1 and p21 in colorectal cancer (Figure 6J). A high level of BRIX1 combined with a low level of p21 could predict a worse prognosis more significantly (Figure 6K). Furthermore, both univariate and multivariate analyses indicated that BRIX1 served as a prognostic factor in colorectal cancer (Figure 6L; Table S4, Supporting Information). Finally, we validated the clinical relevance of BRIX1 through the Cancer Genome Atlas (TCGA) database. Both mRNA and protein levels of BRIX1 were upregulated in various cancerous tissues compared to normal tissues (Figure S4A–H, Supporting Information), and higher levels of BRIX1 were associated with worse prognoses in different types of cancer (Figure S4I–P, Supporting Information). Taken together, the above findings demonstrate that the high level of BRIX1, which facilitates ribosome biogenesis and restricts p53 activity, is crucial for the progression of cancer, whereas depleting BRIX1 suppresses cancer development by activating p53 through nucleolar stress (Figure 6M).

### 2.7 Delivery of Therapeutic BRIX1 siRNA via iRGD‐Modified Exosomes Suppresses Colorectal Cancer

Based on our findings, targeting BRIX1 might be a potential strategy for cancer therapy by triggering nucleolar stress. Therapeutic RNAi has emerged as a powerful approach that can be utilized to treat a variety of diseases, including cancer, by effectively inhibiting the expression of any particular gene.^[^ ^46^, ^47^ ^]^ However, the use of RNAi has faced numerous challenges, such as siRNA instability, insufficient cell or tissue penetration, and the induction of immune response. Exosomes are natural nanoparticles ranging from 30 to 150 nm in diameter and have shown considerable potentials as vehicles for drug delivery.^[^ ^48^, ^49^, ^50^ ^]^ For instance, exosomes display superior biocompatibility because they do not carry unwanted exogenous factors that stimulate the immune system. In addition, the surface expression of CD47 prevents the clearance of exosomes by monocytes and macrophages.^[^ ^51^ ^]^ Recently, a number of therapeutic exosomes that deliver RNAi drugs have been reported to inhibit the growth of cancer.^[^ ^51^, ^52^, ^53^ ^]^ Thus, we attempted to establish an exosome‐based delivery system loaded with the siRNA against BRIX1 for the treatment of cancer.

**Figure 7:**
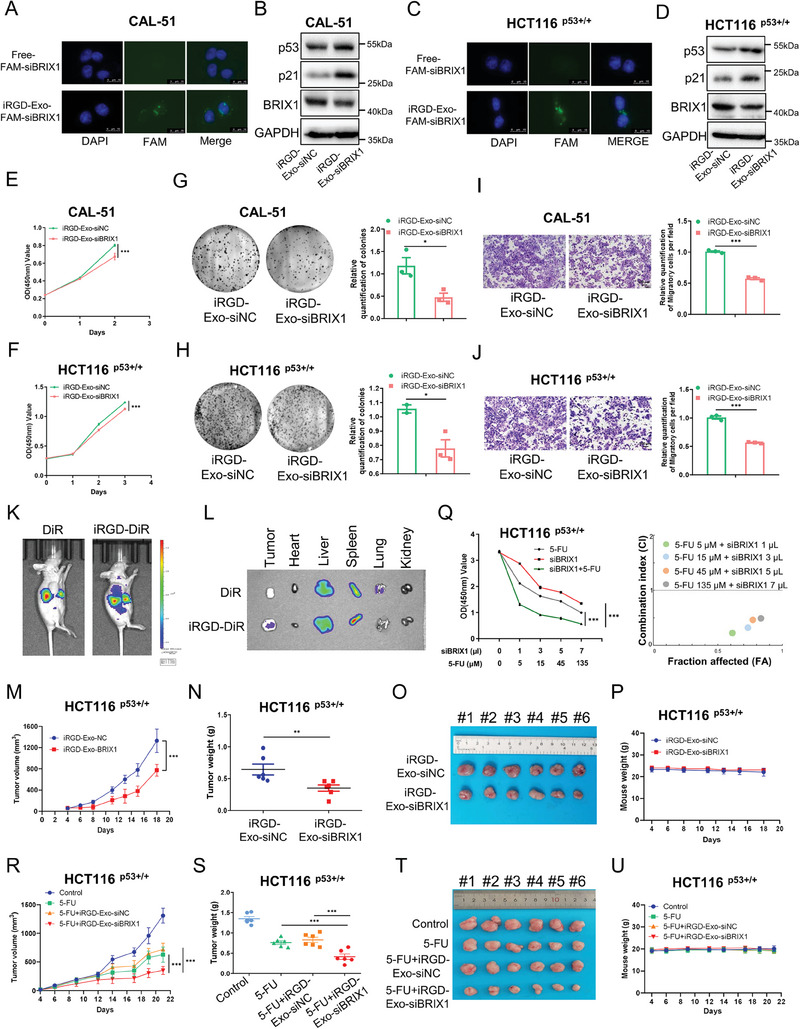
Delivery of therapeutic BRIX1 siRNA via iRGD‐decorated exosomes suppresses colorectal cancer in vitro and in vivo. A, C) iRGD‐decorated exosomes transport FAM‐labeled siBRIX1 into cancer cells. CAL‐51 and HCT116 ^p53+/+^ cells were incubated with FAM‐siBRIX1 or iRGD‐Exo‐FAM‐siBRIX1 for 8 h, followed by fluorescence microscopy. FAM‐siBRIX1, green; DAPI, blue for nucleus; scale bars, 10 µm. (B, D) iRGD‐Exo‐siBRIX1 activates p53. CAL‐51 B) and HCT116 ^p53+/+^ D) cells were incubated with the indicated exosomes for 48 h, followed by IB analysis. E‐J) iRGD‐Exo‐siBRIX1 inhibits the growth E, F), colony formation G, H), and migration I, J) in CAL‐51 and HCT116 ^p53+/+^ cells. CAL‐51 and HCT116 ^p53+/+^ cells were incubated with the indicated exosomes for 24 h, followed by the cell viability assay, colony formation assay, or cell migration assay. (K, L) iRGD increases the enrichment of exosomes in tumor area. DiR‐labeled exosomes were injected intravenously into BALB/c nude mice bearing xenograft tumors derived from HCT116 ^p53+/+^cells and analyzed by in vivo fluorescence imaging. M‐P) iRGD‐Exo‐siBRIX1 inhibits the growth of xenograft tumors derived from HCT116 ^p53+/+^ cells. Q) The combination of siBRIX1 and 5‐FU synergistically inhibits cancer cell growth. HCT116 ^p53+/+^cells were treated with combinations of agents as indicated for 48 h, followed by a cell viability assay and Chou‐Talalay analysis. R‐U) The combination of iRGD‐Exo‐siBRIX1 and 5‐FU coordinately suppresses the growth of xenograft tumors derived from HCT116 ^p53+/+^ cells. Data are presented as mean ± SD, *n* = 6. **p < 0.01, ***p < 0.001.

First, we fused the tissue‐penetrating internalizing RGD (iRGD) peptide, which may direct exosomes to tumors by binding to both αv integrins and neuropilin‐1,^[^ ^54^ ^]^ to the N‐terminus of the exosomal surface protein LAMP2B. The plasmids encoding the fused iRGD‐LAMP2B protein were stably transfected into HEK293T cells, serving as donor cells to produce engineered exosomes with the expression of iRGD on the surface (Figure S5A, Supporting Information). The morphology of purified exosomes was validated by transmission electron microscopy (TEM) (Figure S5B, Supporting Information) and nanoparticle tracking analysis (NTA) (Figure S5C, Supporting Information). In addition, the exosomal markers TSG101, CD81, and CD9 were exclusively detected in exosomes, while the mitochondrial protein COXIV served as a reference for comparison (Figure S5D, Supporting Information). The expression of the ligand of iRGD was confirmed in colorectal and breast cancer cells (Figure S5E, Supporting Information). iRGD decoration increased the uptake of PKH67‐labeled exosomes by cancer cells (Figure S5F, Supporting Information). Next, FAM‐tagged siRNAs were loaded into iRGD‐decorated exosomes by electroporation. CAL‐51 and HCT116 ^p53+/+^ cells were treated with these exosomes for 8–12 h and observed under a fluorescence microscope. Our results showed that siRNAs loaded within exosomes (iRGD‐Exo‐FAM‐siRNAs), but not free FAM‐siRNAs, could be taken by cancer cells (**Figure** **7**A,C). Also, iRGD‐Exo‐siBRIX1 markedly elevated the levels of p53 and p21 by knocking down the expression of BRIX1 in both cell lines (Figure 7B,D). Additionally, we tested whether iRGD‐Exo‐siBRIX1 inhibited the growth and migration of cancer cells. As anticipated, treatment of CAL‐51 and HCT116 ^p53+/+^ cells with iRGD‐Exo‐siBRIX1 significantly reduced their proliferation (Figure 7E,F), colony formation (Figure 7G,H), and migration (Figure 7I,J). These results indicate that the engineered exosomes can serve as vehicles for the delivery of BRIX1 siRNA into cancer cells.

To test whether iRGD‐decorated exosomes target tumors, we administered intravenously the exosomes labelled with DiR, a lipophilic fluorescent dye, to xenograft mice. Although exosomes were predominantly localized in livers and spleens as previously described,^[^ ^55^ ^]^ iRGD could markedly increase the accumulation of exosomes in the xenograft tumor (Figure 7K,L). Next, we investigated whether iRGD‐Exo‐siBRIX1 suppressed tumor growth in vivo. The siRNAs used in the following experiments underwent 2′‐O‐methyl modifications that enhanced their stability and binding affinity and diminished immune activation.^[^ ^46^ ^]^ BALB/c nude mice were subcutaneously inoculated with HCT116 ^p53+/+^ cells and iRGD‐Exo‐siBRIX1 or iRGD‐Exo‐siNC was then intravenously administered three times a week. Our results revealed that iRGD‐Exo‐siBRIX1 significantly inhibited the growth rate (Figure 7M), weight (Figure 7N), and size (Figure 7O) of xenograft tumors, while no significant body weight loss was observed (Figure 7P). IB analysis of these tumor tissues revealed that iRGD‐Exo‐siBRIX1 induced the expression of p53 and p21 in vivo (Figure S5G, Supporting Information). Moreover, we sought to investigate if iRGD‐Exo‐siBRIX1 combined with chemotherapy could coordinately suppress colorectal cancer. To test this hypothesis, we first determined if knockdown of BRIX1 enhanced the sensitivity of HCT116 ^p53+/+^ cells to 5‐FU. The results showed that depletion of BRIX1 reduced the half‐maximal inhibitory concentration (IC50) of 5‐FU (Figure S5H, Supporting Information) and markedly inhibited cell growth in combination with 5‐FU (Figure S5I, Supporting Information). In addition, the combination of siBRIX1 and 5‐FU exhibited a strong synergistic effect on cell viability because the combination indexes (CI) were around or less than 0.5 (Figure 7Q). Then, we evaluated the combination effect of iRGD‐Exo‐siBRIX1 and 5‐FU in vivo. Nude mice bearing xenograft tumors were randomly divided into four groups for different treatments, including saline, 5‐FU, 5‐FU combined with iRGD‐Exo‐siNC, and 5‐FU combined with iRGD‐Exo‐siBRIX1. As expected, 5‐FU administration suppressed the growth of xenograft tumors (Figure 7R–T). Remarkably, the combination of 5‐FU and iRGD‐Exo‐siBRIX1 resulted in a more pronounced anti‐tumor effect in xenograft mice, as evidenced by the marked reduction of tumor growth rate, weight, and size (Figure 7R–T). The potential drug‐related adverse events were tolerable, as the average weight of the treated mice was comparable to that of the control mice (Figure 7U). Altogether, these results demonstrate that targeted inhibition of BRIX1 via iRGD‐modified exosomes can effectively suppress the growth of colorectal tumors and enhance their sensitivity to chemotherapy.

## 3 Discussion

A high rate of ribosome biogenesis is essential for the rapid growth and proliferation of cancer cells. The impairment of this process leads to the translocation of RPs from the nucleolus to the nucleoplasm, thereby preventing MDM2‐induced proteasomal degradation of p53.^[^ ^8^, ^9^, ^10^ ^]^ In this study, we identified that BRIX1 was a key regulator of ribosome biogenesis and nucleolar stress. BRIX1 plays an oncogenic role through at least two mechanisms. First, BRIX1 bound to both BOP1 and PES1 and facilitated the formation of the PeBoW complex. This was mainly required for the processing of 32S pre‐rRNA (Figure 2). In addition, BRIX1 interacted with both RPL5 and RPL11, thereby preventing the interactions between the RPs and MDM2. This led to an increase in MDM2‐induced ubiquitination and degradation of p53 (Figure 4A–K; Figure S3C,D, Supporting Information). These results revealed that BRIX1 promoted the growth and progression of cancer by facilitating ribosome biogenesis and restricting p53 activity. More importantly, depletion of BRIX1 triggered nucleolar stress by impairing the processing of pre‐rRNA (Figure 2A–D,L; Figure S2E, Supporting Information), resulting in increased interactions between RPL5/RPL11 and MDM2 and the consequent stabilization and activation of p53 (Figures 1C–L and 3A–H). Finally, our results demonstrated that targeted inhibition of BRIX1 by therapeutic exosomes efficiently enhanced 5‐FU chemotherapy and suppressed the growth of colorectal cancer with wild‐type p53 (Figure 7; Figure S5, Supporting Information).

It was noticed that BRIX1 could act through a p53‐independent fashion, as knockdown of BRIX1 slightly but somewhat significantly inhibited the growth, colony formation, and migration of p53‐null colorectal cancer cells (Figure 5). There are several mechanisms that may account for these effects. It is possible that nucleolar stress‐induced ribosome‐free RPs prevent the recruitment of c‐Myc and its co‐factor TRRAP on the target gene promotes^[^ ^16^ ^]^ or increase the turnover of c‐Myc mRNA through the microRNA/RISC complex.^[^ ^17^, ^18^, ^19^ ^]^ In addition, several RPs, such as RPL5, RPL11, and RPS14, bind to the transactivation domain of TAp73, thus preventing the inhibition of TAp73 by MDM2,^[^ ^15^ ^]^ while RPL26 associates with the 3′ untranslated region (3′ UTR) of TAp73 mRNA to boost its translation.^[^ ^56^ ^]^ Moreover, ribosome‐free RPs have also been shown to regulate several other cellular signals that are involved in DNA damage repair, angiogenesis, and therapeutic response.^[^ ^10^ ^]^ Additional investigations may be needed to elucidate the action of BRIX1 in p53‐null or ‐mutated tumors.

There is mounting evidence to suggest that triggering nucleolar stress could be a potent approach for cancer treatment. First, ablation of RPs or nucleolar proteins, which induces nucleolar stress, can efficiently inhibit the growth of cancer cells in vitro and in vivo by activating p53.^[^ ^8^, ^29^, ^30^ ^]^ In addition, numerous chemotherapeutic drugs have been reported to induce nucleolar stress. Act D is used for the treatment of Hodgkin's lymphoma and neuroblastoma by inhibiting the function of all three RNA Pols, while a low dose of Act D can preferentially impede RNA Pol I activity, leading to nucleolar stress.^[^ ^12^ ^]^ DNA damage‐inducing agents, such as 5‐FU, doxorubicin, camptothecin, and platinum drugs, also impair rRNA synthesis at both transcriptional and post‐transcriptional levels.^[^ ^20^, ^21^ ^]^ Recently, the PARP inhibitor Olaparib was found to repress the production of rRNA possibly by reducing ADP‐ribosylation and nucleolar localization of DDX21, leading to RPL5/RPL11‐depednent activation of p53.^[^ ^22^, ^57^ ^]^ Interestingly, recent studies revealed that a low dose of Act D or targeted inhibition of the nucleolar proteins, WDR75 and UTP11, promoted p53‐ or NRF2‐dependent ferroptosis.^[^ ^31^, ^32^ ^]^ Considering that the induction of ferroptosis can sensitize resistant cancer cells to chemotherapy, the combination of nucleolar stress inducers and chemotherapeutic agents could be an effective strategy for cancer therapy.^[^ ^58^ ^]^ However, the development of compounds that selectively impair ribosome biogenesis has been challenging.^[^ ^23^, ^24^, ^25^ ^]^ CX‐5461 impedes the recruitment of SL1, an essential component of the RNA Pol I initiation complex, on the rDNA promoter.^[^ ^23^, ^24^ ^]^ BMH‐21 associates with GC‐rich rDNA genes and suppresses RNA Pol I activity by promoting the proteasomal degradation of the large catalytic subunit RPA194.^[^ ^25^ ^]^ While both compounds can induce nucleolar stress, they have also been shown to trigger DNA or chromatin damage possibly because of their ability to directly associate with DNA or DNA repair components.^[^ ^26^, ^27^, ^28^ ^]^ Notedly, our results revealed that depleting BRIX1 impaired pre‐rRNA processing (Figure 2) and perturbed nucleolar architecture as indicated by the relocation of NPM1 (Figure S2E, Supporting Information). This resulted in nucleolar stress without causing DNA damage (Figure 1M), leading to RPL5/RPL11‐dependent p53 activation. In contrast, excessive BRIX1 prevented p53 activation by trapping RPL5 and RPL11. Therefore, our study as presented here suggests that BRIX1 is an alternative target molecule for the selective induction of nucleolar stress.

As our knowledge of cancer grows, a plethora of tumor markers or potential therapeutic targets have been discovered. However, the primary obstacle remains the translation of the target molecules into clinical practice. A significant portion of oncoproteins present challenges for drug targeting due to the lack of suitable compound‐binding sites. Recently, small nucleic acid drugs have gained tremendous attentions, as therapeutic RNAi technically offers new avenues for targeted inhibition of previously undruggable molecules. For example, siRNA specific to oncogenic KRAS ^G12D^ delivered by engineered exosomes suppress pancreatic cancer in multiple mouse models and significantly increase overall survival.^[^ ^51^ ^]^ In our study, we developed iRGD‐decorated exosomes loaded with siRNA against BRIX1 and verified that these exosomes could transport siBRIX1 to tumor sites (Figure 7K,L) and increase the uptake of siBRIX1 by cancer cells (Figure 7A,C). Noteworthily, our results demonstrated that iRGD‐Exo‐siBRIX1 effectively suppressed the growth of colorectal cancer and enhanced the efficacy of 5‐FU chemotherapy in vivo (Figure 7M–U). While iRGD has been reported for use in nanomedicine,^[^ ^59^ ^]^ the tumor‐targeting potential of exosomes could potentially be further improved. For instance, the human epidermal growth factor receptor 2 (HER2) and folate receptor (FR) are highly expressed in colorectal and breast cancers. Modifications of exosomes with HER2 affinity peptides^[^ ^60^ ^]^ or folates^[^ ^61^ ^]^ could be alternative strategies. Also, surface decoration of exosomes with CD47 extends their blood circulation time.^[^ ^62^ ^]^ In addition, exosomes coated with the M2 macrophage binding peptide, M2pep, can specifically target tumor‐associated macrophages.^[^ ^63^ ^]^

In conclusion, our study identifies BRIX1 as an oncoprotein that is overexpressed in colorectal and breast cancers, correlating with unfavorable prognoses. BRIX1 facilitates the processing of pre‐rRNA by interacting with the PeBoW complex and represses p53 activity by sequestering RPL5 and RPL11. In contrast, depletion of BRIX1 activates p53 to suppress the growth of cancer by triggering nucleolar stress. Finally, our results demonstrate that utilizing an exosome‐based delivery system loaded with siRNA specific to BRIX1 could be a promising approach for cancer treatment.

## 4 Experimental Section

### Cell Culture and Transient Transfection

Human colorectal cancer cell lines, HCT116 ^p53+/+^, HCT116 ^p53−/−^, RKO, and SW480, breast cancer cell lines, CAL‐51, MCF‐7, and MDA‐MB‐231, and embryonic kidney cell line 293T were cultured in DMEM (Dulbecco's modified Eagle's medium) supplemented with 10% FBS (Yeasen, Shanghai, China), 100 units mL^−1^ penicillin, and 100 µg mL^−1^ streptomycin (BasalMedia, Shanghai, China). All the cell lines were mycoplasma free and authenticated by PCR analysis. Cells were seeded on the plates at an optimal density 12–24 h before transfection. Transfection was conducted using Hieff Trans liposome transfection reagent (Yeasen) according to the manufacturer's protocol.

### Plasmids and Antibodies

The cDNAs of BRIX1, BOP1, PES1, and WDR12 were amplified using the designed primers (Table S5, Supporting Information). Subsequently, the cDNA sequences were cloned into the Flag‐pCDNA3.1, Myc‐pCDNA3.1, and HA‐pCMV vector. BRIX1 cDNA sequences were ligated into the lentiviral vector to generate pCDH‐Flag‐BRIX1. The plasmids encoding HA‐MDM2, p53, and His‐Ub were described previously.^[^ ^64^ ^]^ The anti‐Flag (Cat. No. F1804, Sigma‐Aldrich, St Louis, MO, USA), anti‐Myc (Cat. No. 60003‐2‐Ig, Proteintech), anti‐HA (Cat. No. 2367, Cell Signaling Technology, Danvers, MA, USA), anti‐BRIX1 (Cat. No. 17295‐1‐AP, Proteintech), anti‐p53 (Cat. No. sc‐126, DO‐1, Santa Cruz Biotechnology), anti‐MDM2 (Cat. No. ab16895, 2A10, Abcam), anti‐Phospho‐Histone H2A.X (Ser139) (Cat. No. 9718, Cell Signaling Technology), anti‐GAPDH (Cat. No. 60004‐1‐Ig, Proteintech), anti‐Tubulin (Cat. No. 66031‐1‐Ig, Proteintech), anti‐RPL5 (Cat. No. ab86863, Abcam), anti‐RPL11 (Cat. No. ab79352, Abcam), anti‐p21 (Cat. No. 2947, Cell Signaling Technology), anti‐NPM1 (Cat. No. sc‐271737, Santa Cruz Biotechnology), anti‐BOP1 (Cat. No.28366‐1‐AP, Proteintech), anti‐TSG101 (Cat. No. 28283‐1‐AP, Proteintech), anti‐CD81 (Cat. No. sc‐166029, Santa Cruz Biotechnology), anti‐CD9 (Cat. No. sc‐13118, Santa Cruz Biotechnology), anti‐COXIV (Cat. No. 11242‐1‐AP, Proteintech), and anti‐αVβ3 (Cat. No. bs‐1310R, Bioss) were commercially purchased. The secondary antibodies used were HRP‐conjugated affinipure goat anti‐rabbit IgG (Cat. No. SA00001‐2, Proteintech) and anti‐mouse IgG (Cat. No. SA00001‐1, Proteintech). Proteins were visualized with the ECL chemiluminescence reagent (Yeasen).

### Reverse Transcription and Quantitative Real‐Time PCR

Total RNA was isolated from cells by RNAiso Plus (Takara, Japan) following the manufacturer's protocol, and cDNA was synthesized using Hiscript III qRT SuperMix (Vazyme). Quantitative PCR (qPCR) was carried out using SYBR qPCR Master Mix according to the manufacturer's protocol (Vazyme). The relative expression levels of mRNAs were calculated using the comparative Ct method normalized to GAPDH. The primers for quantitative PCR (qPCR) are listed in (Table S6, Supporting Information).

### RNA‐Sequencing

The CAL‐51 cells were transfected with siNC or siBRIX1 for 48 h and total RNA was isolated by RNAiso Plus (Takara, Japan). RNA sequencing was provided by OE Biotech Co., Ltd (shanghai, China).

### Immunoblotting

Proteins were extracted using RIPA buffer [50 mM Tris/HCl (pH8.0), 150 mM NaCl, 1% Triton X‐100, 0.5% Sodium deoxycholate, 0.1%SDS, 0.2 mM phenylmethylsulfonyl fluoride, and 10% protease inhibitor cocktail] and quantified using BCA reagent (Yeasen). Equal amounts of clear cell lysate (20–80 µg) were used for immunoblotting (IB) analysis as described previously.^[^ ^29^ ^]^

### Northern Blotting

Northern blotting was performed as previously described.^[^ ^32^ ^]^ NorthernMax‐Gly Kit (Thermo Fisher Scientific, USA) was used for the Northern blotting assay according to the manufacturer's instructions. Briefly, RNA (30–60 µg) denatured with Glyoxal Load Dye was separated on 1% agarose gel for electrophoresis and transferred to BrightStar‐Plus nylon membrane (Thermo Fisher Scientific, USA). Then, RNA was crosslinked to the membrane by 1.5 J cm^2^ UV exposure and the membrane was pre‐hybridized at 65 °C for 30 m and hybridized with the probe at 42 °C overnight. The next day, the membrane was washed for 10 m at room temperature and 2 m at 42 °C with Low Stringency Wash Solution and blocked with blocking buffer for 30 m. Subsequently, the membrane was incubated with anti‐DIG for 1 h at room temperature (Universal Biotech Co, shanghai) and washed with washing buffer twice for 15 m and with detecting buffer for 5 m. Finally, the membrane was developed with the CDP‐Star (Roche, USA). The probe used in this study was described previously^[^ ^40^ ^]^ and the digoxigenin (DIG)‐labeled probe was synthesized by GENEWIZ (Suzhou, China).

### Immunofluorescence Staining

Cells transfected with siRNAs were fixed with methanol overnight at −20 °C, washed with PBS three times, and blocked with blocking buffer (8% BSA and 0.3% Triton X‐100) for 1 h at room temperature. Cells were then incubated with primary antibodies (anti‐BRIX1, 1:100; anti‐NPM1,1:50 dilution) at 4 °C for overnight. The next day, the cells were washed with PBS and incubated with Fluorescent secondary antibodies (Yeasen) and DAPI (Sigma‐Aldrich). Images were obtained with an inverted fluorescence microscope (Leica, Wetzlar, Germany).

### Immunoprecipitation

Proteins were extracted using lysis buffer [50 mM Tris/HCl (pH7.5), 0.5% Nonidet P‐40 (NP‐40), 1 mM EDTA, 150 mM NaCl, 1 mM dithiothreitol (DTT), 0.2 mM phenylmethylsulfonyl fluoride, and 10% protease inhibitor cocktail] and immunoprecipitation (IP) was performed using antibodies as indicated in the figure legends. Briefly, proteins (0.5–1 mg) were incubated with the indicated antibody at 4 °C for 5 h, followed by the incubation with protein A/G magnetic beads (Santa Cruz Biotechnology) for additional 2 h. The beads containing immunoprecipitated proteins were washed 6–8 times with lysis buffer and analyzed by IB as described above.^[^ ^65^ ^]^

### In Vivo Ubiquitination Assay

HCT116 ^p53−/−^ cells were transfected with plasmids encoding p53, HA‐MDM2, His‐Ub, and BRIX1 siRNA as indicated in the figure legends and treated with MG132 for 4–6 h before being harvested for the in vivo ubiquitination assay as previously described.^[^ ^32^ ^]^ In brief, at 48 h after transfection, cells were harvested and split into two aliquots, one for IB and the other one for the ubiquitination assay. Cell pellets were lysed in buffer I [8 M urea, 0.1 M Na_2_HPO_4_/NaH_2_PO_4_ (pH 8.0), 10 mM Tris‐HCl (pH 8.0), 10 mM β‐mercaptoethanol, and 5 mM Imidazole] and incubated with Ni‐NTA beads (Takara) that capture His‐tagged proteins/complex at room temperature for 4 h. Beads were washed twice with buffer I, then twice with buffer II [8 M urea, 0.1 M Na_2_HPO_4_/NaH_2_PO_4_ (pH 6.3), 10 mM Tris‐HCl (pH 6.3), and 10 mM β‐mercaptoethanol] The captured proteins were eluted and analyzed by IB with the indicated antibodies.

### RNA Interference and Generation of Stable Cell Lines

The small interfering RNAs (siRNA) against BRIX1, RPL5, and RPL11 were synthesized and purified by GenePharma (Shanghai, China). The detailed sequences are provided in (Table S7, Supporting Information). The transfection of siRNAs was performed using Hieff Trans Liposomal transfection reagent following the manufacturer's protocol (Yeasen). All transfections were validated with qPCR or western blot. The specific sequences of shRNA targeting BRIX1 were obtained from Sigma‐Aldrich and cloned into the PLKO.1 vector (Table S8, Supporting Information). The plasmid containing shRNA along with the packaging plasmids, psPAX2 and pMD2.G, were introduced into HEK293T cells. The lentiviral particles were collected 48 h after transfection and then used for cell infection. The stable cells were selected with 1 µg mL^−1^ puromycin.

### Cell Viability Assay

To evaluate the cell viability, 2–4×10^3^ cells were seeded per well in 96‐well culture plates in triplicate at 6–12 h post‐transfection. The Cell Counting Kit‐8 (CCK‐8) reagent (Yeasen) was added to each well at a final concentration of 10%. The mixture was incubated for 2 h at 37 °C, and the absorbance at 450 nm was measured using a microplate reader. For drug combination analysis, the combination indexes (CI) plotted against the fraction affected (FA) were calculated by the CompuSyn software using the Chou‐Talalay method.^[^ ^66^ ^]^ CI indicates the synergistic, additive or antagonistic effect when the value is less, equal to or greater than 1.

### Colony Formation Assay

1×10^3^ cells were plated onto a 6 cm plate 6–18 h after siRNA transfection and cultured for 14 days. The medium was changed every 3 days until colonies were visible. Colonies were then fixed with 100% methanol and stained with 0.2% crystal violet solution at room temperature for 30 m. The number of colonies was determined by ImageJ.

### Cell Cycle Assay

After transfection with siRNAs for 48 h, cells were fixed in ice‐cold 70% ethanol with PBS overnight, treated with 50 µg mL^−1^ RNase A (Sangon) and 0.1% Triton X‐100 (Sangon) in PBS at 37 °C for 30 m, and stained with 50 µg mL^−1^ propidium iodide (PI) (Vazyme) for 30 m in the dark. Finally, the cell cycle was analyzed by flow cytometry (CytoFLEX S, Beckman Coulter, Indianapolis, IN, USA).

### Transwell Cell Migration Assay

For cell migration assay, 5–10 × 10^4^ cells in 200 µL serum‐free medium were seeded in the upper chamber and the lower chamber contained 800 µL 20% FBS culture medium. After culture for 36–48 h at 37 °C, cells on the lower surface of the chamber were fixed with methanol and stained with 0.2% crystal violet (BBI Life Sciences) for 30 m. Migratory cells were counted using a microscope and quantified by ImageJ.

### Apoptosis Assay

Apoptosis assay was performed following the manufacturer's protocol of the apoptosis detection kit (Vazyme). Briefly, cells were washed twice with cold PBS and then re‐suspended in 1 × Binding Buffer at a concentration of 1 × 10^6 ^cells mL^−1^. Cells were incubated with Annexin V‐PE and 7‐aminoactinomycin D (7AAD) for 15 m at room temperature in the dark. In the end, the level of apoptosis was determined by flow cytometry (CytoFLEX S, Beckman Coulter, Indianapolis, IN, USA).

### In Vivo Xenograft Assay

Five‐week‐old female BALB/c nude mice were obtained from Laboratory Animal Science of Fudan University Shanghai Cancer Center. To investigate the function of BRIX1, 5 × 10^6^ HCT116 ^p53+/+^ and HCT116 ^p53−/−^ cells stably expressing shNC or shBRIX1, and PCDH or PCDH‐BRIX1 resuspended in 100 µL serum‐free medium were injected into flank regions of mice. Both tumor volume and weight of mice were measured as indicated in the figure. Tumor volume was calculated with the formula: tumor volume (mm^3^) = (length × width^2^) × 0.52. To investigate the anti‐tumor effect of iRGD‐Exo‐siBRIX1, we injected exosomes into tumor‐bearing mice via the tail vein three times a week. To explore the combined effect of iRGD‐Exo‐siBRIX1 with chemotherapy, tumor‐bearing mice were randomly divided into four groups and respectively administered with saline, 10 mg kg^−1^ 5‐FU, and the combination of 10 mg kg^−1^ 5‐FU with 150 µg exosomes containing 3.75 µg siNC (iRGD‐Exo‐siNC) or with 150 µg exosomes containing 3.75 µg siBRIX1 (iRGD‐Exo‐siBRIX1). Saline and 5‐FU were administered intraperitoneally, while engineered exosomes were administered intravenously. When the tumors reached an appropriate volume, the nude mice were sacrificed and the tumors were harvested, weighed, and photographed. All studies were approved by the Animal Welfare Committee of Fudan University Shanghai Cancer Center, and all animals were treated according to the institutional guidelines.

### Human Breast Cancer and Colorectal Cancer Specimens

A total of five pairs of fresh breast cancer and adjacent normal tissues obtained from the First Affiliated Hospital of China Medical University were used to detect the levels of BRIX1 protein and mRNA. Ninety‐one paraffin‐embedded sections of breast cancer obtained from the First Affiliated Hospital of China Medical University were subjected to IHC analysis. In addition, a total of ten pairs of colorectal cancer and adjacent normal tissues and 62 paraffin‐embedded sections of colorectal cancer used for IHC analysis were obtained from the First Affiliated Hospital of Nanchang University. Informed consent was obtained from all patients, and this study was approved by the Human Research Ethics Committee of the First Affiliated Hospital of China Medical University (AF‐SOP‐07‐1.1‐01/2019‐13) and the First Affiliated Hospital of Nanchang University [(2024)CDYFYYLK(04‐002)].

### Immunohistochemistry

Paraffin‐embedded tissue samples of breast or colorectal cancer tissues were deparaffinized, rehydrated, and treated with Sodium citrate‐EDTA antigen repair solution (cat. No. P0086, Beyotime) for antigen retrieval. Following that, the tissues were incubated with primary antibodies for 1–2 h at room temperature. After extensive washing with PBS, tissues were incubated with a secondary antibody (cat. No. GK500705, GeneTech) at room temperature for 1 h. Subsequently, the sections were treated with 3′‐diaminobenzidine (cat. No. GK500705, GeneTech) for 5 m to show staining signaling and then were counterstained with hematoxylin. The staining density was measured by using a Leica CCD camera DFC420 connected to a Leica DM IRE2 microscope (Leica Microsystems Imaging Solutions Ltd.).

### Isolation and Purification of Exosomes

Exosomes were collected from the culture medium of HEK293T cells that expressed a plasmid encoding the iRGD‐LAMP2B fusion protein. Samples were centrifuged at 300×g for 10 m to remove cells and larger debris. The supernatant was collected and centrifuged at 10 000×g for 30 m to remove cell debris and other contaminants. The resulting supernatant was centrifuged at 100 000×g for 70 m to pellet exosomes along with any contaminating proteins. The pellet was washed with PBS and then centrifuged again at 100 000 x g for 70 m. The exosomes were stored at –80 °C. Umibio Co., Ltd (Shanghai) provided the service of transmission electron microscopy (TEM) and nanoparticle tracking analysis (NTA).

### SiRNA Loading into Exosomes

The siRNA for BRIX1 (siBRIX1‐2) with 2′‐O‐methyl modification was purchased from GenePharma (Shanghai, China). Combine the siRNA solution with the exosome suspension (with a ratio of 1 µg siRNAs to 4 µg exosomes) in an electroporation cuvette. Electroporation was conducted using settings of 400 V and 125 µF. The encapsulation efficiency of siRNA in exosomes is ≈10%. After electroporation, the mixture was incubated on ice for 5 m and then centrifuged at 100 000 g for 60 m to remove free siRNAs and pellet the exosomes.

### Statistical Analysis

All in vitro experiments were conducted in biological triplicate. The animal cohorts for in vivo experiments were randomly allocated to different groups. Differences between two or more groups were analyzed by the Student's t‐test or one‐way ANOVA. Statistical analyses were conducted using GraphPad Prism 8.0 and presented as means ± the standard deviation (SD). Kaplan‐Meier plot method and log‐rank tests were used to analyze significant differences in patient survival. Multivariate Cox proportional hazard models generated hazard ratios with 95% confidence intervals. Asterisks denote statistical significance: *p < 0.05; **p < 0.01; ***p < 0.001.

## Conflict of Interest

The authors declare no conflict of interest.

## Author Contributions

Y.G., Q.H., and T.H. contributed equally to this work. Y.G., Q.H., and T.H. conducted the experiments and analyzed the data. J.T., Q.Y., and H.Z. conducted part of experiments. B.G., Y.L., and Z.X. constructed and purified engineered exosomes. P.L., L.Y., and Y.X. provided materials and analyzed the data. Y.‐Z.J. and Z.‐M.S. provided materials and instructions. Q.H., J.D., J.C., and X.Z. conceived, designed, and supervised the study and analyzed the data; Y.G. and X.Z. wrote and revised the manuscript.

## Ethics Approval and Consent to Participate

The present study was approved by the ethics committee of the participating institutions.

## Supporting information



Supporting Information

## Data Availability

The data that support the findings of this study are available from the corresponding author upon reasonable request.
