# Supplementary material for: Targeting BRIX1 via Engineered Exosomes Induces Nucleolar Stress to Suppress Cancer Progression
Source: Adv Sci (Weinh). 2024 Oct 30;11(47):2407370. doi: 10.1002/advs.202407370 (PMC11653718; doi:10.1002/advs.202407370)
Supplement: Supplementary file 1 — Supporting Information [file ADVS-11-2407370-s001.pdf]

ADVANCED SCIENCE
Open Access

Supporting Information

for *Adv. Sci.*, DOI 10.1002/advs.202407370

Targeting BRIX1 via Engineered Exosomes Induces Nucleolar Stress to Suppress Cancer Progression

Yu Gan, Qian Hao, Tao Han, Jing Tong, Qingya Yan, Hongguang Zhong, Bo Gao, Yanan Li, Zhisheng Xuan, Pengfei Li, Litong Yao, Yingying Xu, Yi-Zhou Jiang, Zhi-Ming Shao, Jun Deng, Jiaxiang Chen* and Xiang Zhou**

Supplementary Information

Targeting BRIX1 via engineered exosomes induces nucleolar stress to suppress cancer progression

Yu Gan[1,2,#], Qian Hao[1,2,#], Tao Han[3,#], Jing Tong[1,2], Qingya Yan[3], Hongguang Zhong[4,5], Bo Gao[6], Yanan Li[6], Zhisheng Xuan[6], Pengfei Li[7], Litong Yao[8], Yingying Xu[8], Yi-Zhou Jiang[2,9], Zhi-Ming Shao[2,9], Jun Deng[4,5,*], Jiaxiang Chen[10,*], and Xiang Zhou[1,2,9,11,*]

[1] Fudan University Shanghai Cancer Center and Institutes of Biomedical Sciences, Fudan University, Shanghai 200032, China

[2] Department of Oncology, Shanghai Medical College, Fudan University, Shanghai 200032, China

[3] Institutes of Health Central Plains, Xinxiang Key laboratory for Molecular Oncology, Xinxiang Medical University, Xinxiang 453003, Henan, China

[4] Department of Oncology, First Affiliated Hospital of Nanchang University, Nanchang 330006, Jiangxi, China

[5] Jiangxi Key Laboratory for Individualized Cancer Therapy, Nanchang 330006, Jiangxi, China

[6] Umibio Co. Ltd., Shanghai 201210, China

[7] Laboratory of Animal Center, Medical Experiment Center, Shaanxi University of Chinese Medicine, Xianyang 712046, China

[8] Department of Breast Surgery, the First Hospital of China Medical University, Shenyang 110001, Liaoning, China

[9] Key Laboratory of Breast Cancer in Shanghai, Department of Breast Surgery, Fudan University Shanghai Cancer Center, Fudan University, Shanghai, 200032, China

[10] Department of Physiology, School of Basic Medical Sciences, Jiangxi Medical College, Nanchang University, Nanchang 330006, P.R. China

[11] Shanghai Key Laboratory of Medical Epigenetics, International Co-laboratory of Medical Epigenetics and Metabolism (Ministry of Science and Technology), Institutes of Biomedical Sciences, Fudan University, Shanghai 200032, China

[#] Equal contribution

[*] Correspondence:

Jun Deng, Email: dengjun19871106@ncu.edu.cn

Jiaxiang Chen, Email: chenjiaxiang@ncu.edu.cn

Xiang Zhou, Email: xiangzhou@fudan.edu.cn

Running title: Inhibition of BRIX1 activates the nucleolar stress-p53 pathway

Supplementary Figure Legends

Figure S1. Ablation of BRIX1 induces p53 activation. Related to Figure 1.

(A) Knockdown of BRIX1, DHX35, EXOSC6, EXOSC7, LSM6, and PPAN induces the level of p21 in HCT116 $^{p53+/+}$ cells. HCT116 $^{p53+/+}$ cells were transfected with control or the indicated siRNAs for 48 h, followed by RT-qPCR analysis. (B) Knockdown of BRIX1 has no significant impact on the mRNA level of p53. HCT116 $^{p53+/+}$ cells were transfected with control or BRIX1 siRNAs for 48 h, followed by RT-qPCR analysis. (C, D) Knockdown of BRIX1 exerts no influence on the level of mutant p53. SW480 (C) and MDA-MB-231 (D) cells were transfected with control or BRIX1 siRNAs for 48 h, followed by IB analysis. (E, F) Knockdown of BRIX1 inhibits the growth of cancer cells with mutant p53. SW480 (E) and MDA-MB-231 (F) cells were transfected with control or BRIX1 siRNAs for 6-12 h and seeded in 96-well plates for a cell viability assay.

Figure S2. BRIX1 promotes pre-rRNA processing through the PeBoW complex. Related to Figure 2.

(A) Analysis of protein-protein interacting network of BRIX1 through the STRING database. (B, C) There is no interaction observed between BRIX1 and WDR12. Cells were transfected with the indicated plasmids, followed by co-IP-IB analysis. (D) The protein levels of BOP1 are not affected by BRIX1 knockdown. (E) BRIX1 knockdown disrupts the nucleolar localization of NPM1. Cells were transfected with control or BRIX1 siRNA, followed by IF staining. (F, G) knockdown of BRIX1 induces apoptosis in CAL-51 and MCF-7 cells by flow cytometric analysis. ***$p < 0.001$.

Figure S3. Overexpression of BRIX1 impairs p53 activation in response to nucleolar stress. Related to Figure 4.

(A, B) Ectopic expression of BRIX1 has no effect on the expression of p53. CAL-51 (A) and HCT116 $^{p53+/+}$ cells (B) were transfected with plasmids as indicated, followed by IB analysis and quantification with ImageJ. (C, D) Ectopic BRIX1 diminishes the expression of p53 and p21 in HCT116 $^{p53+/+}$ cells when treated with DDP (C) or 5-FU (D). (E) Ectopic BRIX1 has no effect on the expression of p53 and p21 in HCT116 $^{p53+/+}$ cells when treated with Nutlin-3. (F, G) Ectopic BRIX1 inhibits Act D-induced apoptosis. CAL-51 (F) and HCT116 $^{p53+/+}$ (G) cells stably overexpressing an empty vector or BRIX1 were treated with 10 nM Act D for 24 h. Cell apoptosis was analyzed by flow cytometry. (H) Overexpression of BRIX1 does not significantly affect the growth of HCT116 $^{p53-/-}$ cells. Cells were transfected with plasmids as indicated and treated with Act D (10 nM), followed by a cell viability assay. (I) Overexpression of BRIX1 exerts no significant influence on the colony-forming ability of HCT116 $^{p53-/-}$ cells. Cells stably overexpressing control or BRIX1-encoding plasmid were treated with Act D (10 nM), followed by a colony formation assay. *$p < 0.05$, ***$p < 0.001$.

Figure S4. Clinical relevance of BRIX1 levels in various cancers. Related to Figure 6.
(A-D) BRIX1 mRNA levels are elevated in multiple human cancers, including breast cancer (A), colon cancer (B), hepatocellular carcinoma (C), and lung adenocarcinoma (D). (E-H) BRIX1 protein levels are elevated in multiple human cancers, including breast cancer (E), colon cancer (F), hepatocellular carcinoma (G), and lung adenocarcinoma (H). (I-P) Higher levels of BRIX1 correlate with worse prognoses in multiple human cancers, including breast cancer (I), colon cancer (J), lung adenocarcinoma (K), hepatocellular carcinoma (L), ovarian cancer (M), esophageal adenocarcinoma (N), head and neck squamous cell carcinoma (O), kidney renal papillary cell carcinoma (P).

Figure S5. Construction and characterization of engineered exosomes. Related to Figure 7.
(A) A schematic illustration of the construction of iRGD-Exo-siBRIX1. (B) The representative TEM image of exosomes. Scale bar: 200 nm. (C) Particle size distribution of exosomes. (D) Biomarkers of purified exosomes were determined by IB analysis using antibodies as indicated. (E) The expression of integrin αvβ3 in HCT116 $^{p53+/+}$, RKO, CAL-51, and MCF-7 cells was assessed by IB analysis. (F) Representative fluorescence images of in vitro internalization of PKH67-labeled exosomes after 4 h incubation with HCT116 $^{p53+/+}$ cells. (G) iRGD-Exo-siBRIX1 induces the expression of p53 and p21 in tumors derived from HCT116 $^{p53+/+}$ cells. The tumors were homogenized and lysed for IB analysis. (H) Depleting BRIX1 reduces the half-maximal inhibitory concentration (IC50) of 5-FU. (I) BRIX1 siRNA and 5-FU coordinately inhibit cancer cell growth. ***$p < 0.001$.

Figure S1

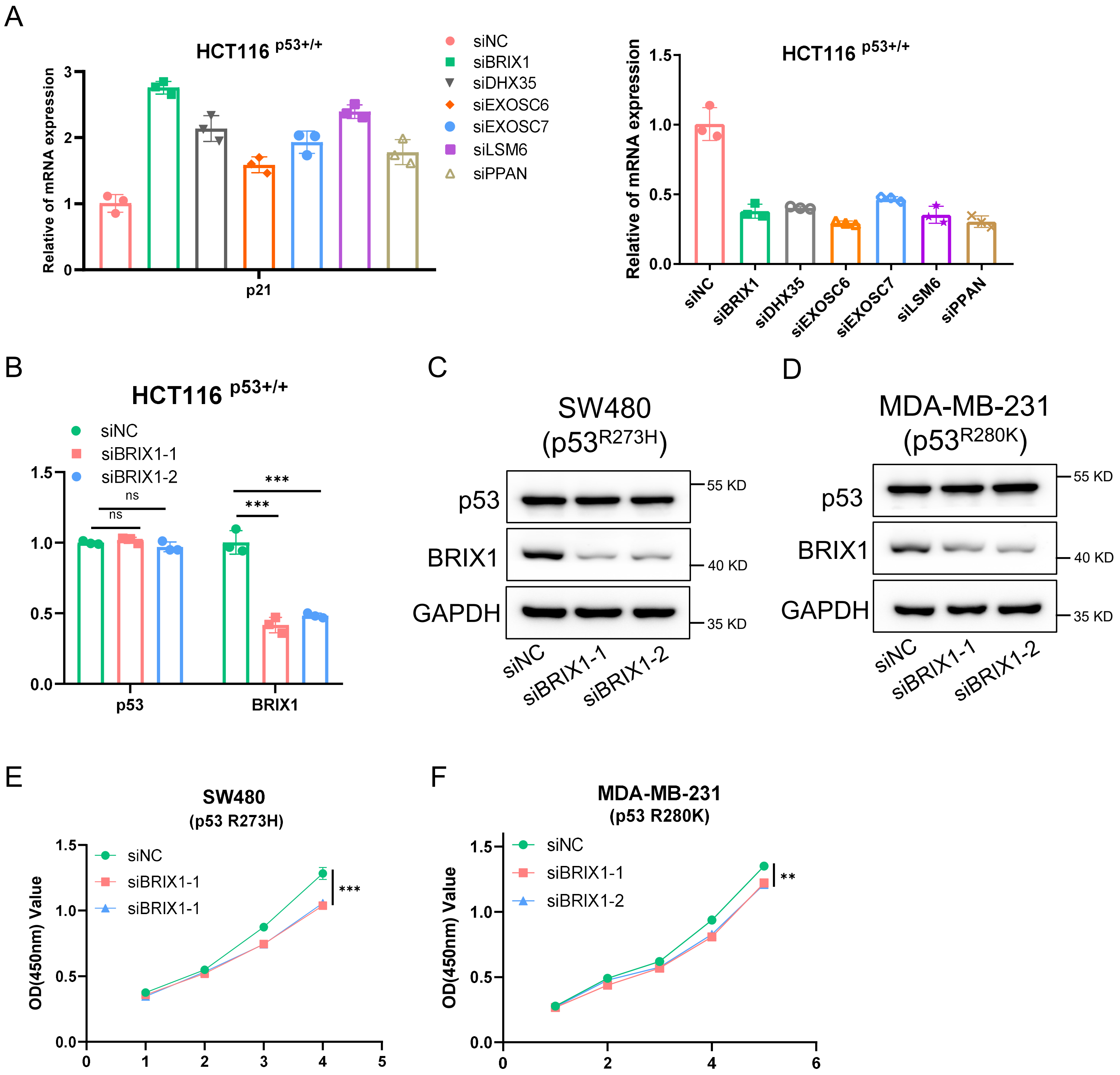
A
HCT116 p53+/+
Relative of mRNA expression
p21
siNC
siBRIX1
siDHX35
siEXOSC6
siEXOSC7
siLSM6
siPPAN
HCT116 p53+/+
Relative of mRNA expression
siNC
siBRIX1
siDHX35
siEXOSC6
siEXOSC7
siLSM6
siPPAN
B
HCT116 p53+/+
siNC
siBRIX1-1
siBRIX1-2
ns
ns
***
***
p53
BRIX1
C
SW480
(p53R273H)
p53
BRIX1
GAPDH
55 KD
40 KD
35 KD
siNC
siBRIX1-1
siBRIX1-2
D
MDA-MB-231
(p53R280K)
p53
BRIX1
GAPDH
55 KD
40 KD
35 KD
siNC
siBRIX1-1
siBRIX1-2
E
SW480
(p53 R273H)
OD(450nm) Value
siNC
siBRIX1-1
siBRIX1-1
***
F
MDA-MB-231
(p53 R280K)
OD(450nm) Value
siNC
siBRIX1-1
siBRIX1-2
**

Figure S2

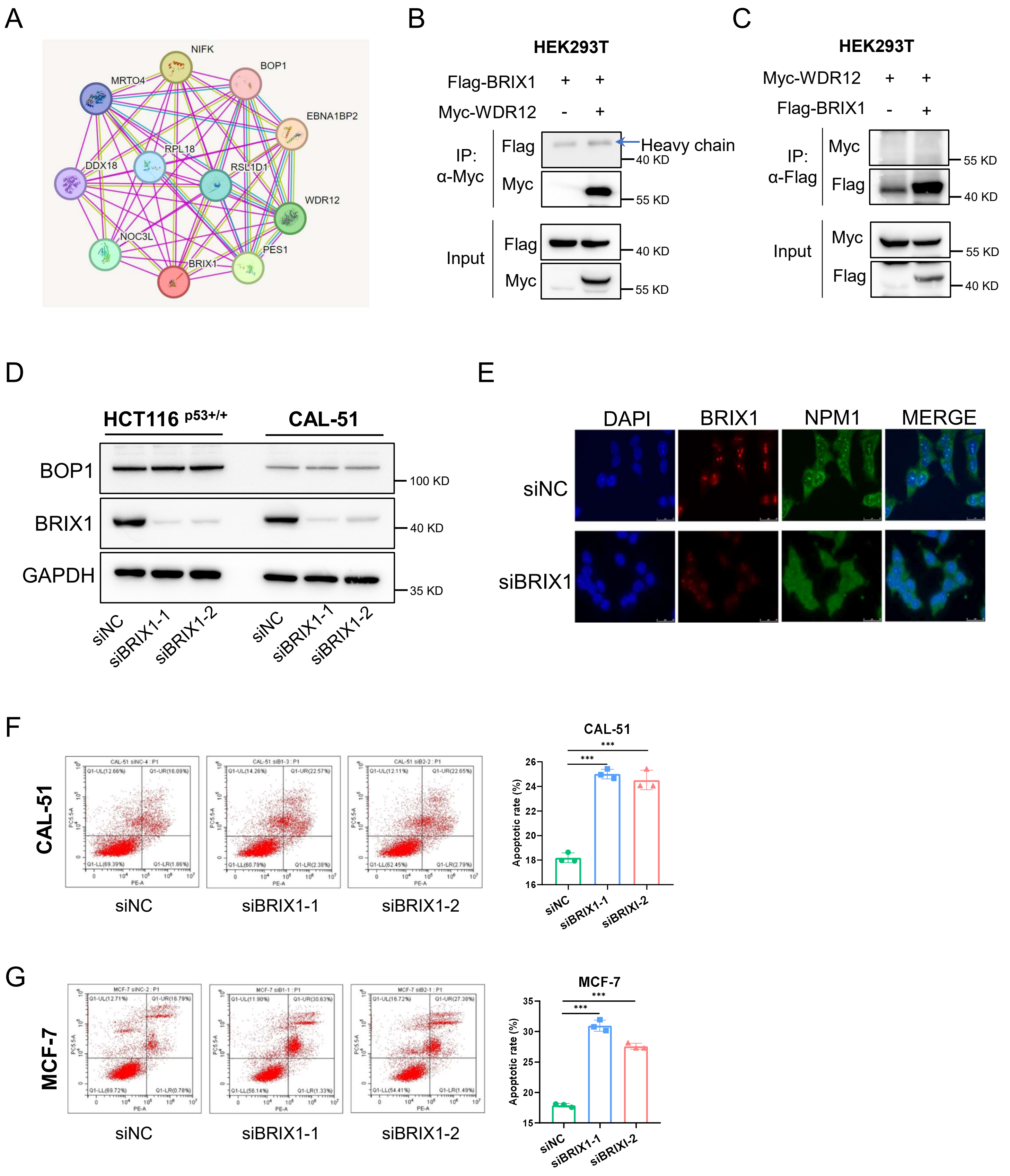
A
NIFK
BOP1
MRTO4
EBNA1BP2
RPL18
DDX18
RSL1D1
WDR12
NOC3L
PES1
BRIX1
B
HEK293T
Flag-BRIX1 + +
Myc-WDR12 - +
IP: α-Myc
Flag
Myc
Heavy chain
40 KD
55 KD
Input
Flag
Myc
40 KD
55 KD
C
HEK293T
Myc-WDR12 + +
Flag-BRIX1 - +
IP: α-Flag
Myc
Flag
55 KD
40 KD
Input
Myc
Flag
55 KD
40 KD
D
HCT116 p53+/+
CAL-51
BOP1
BRIX1
GAPDH
100 KD
40 KD
35 KD
siNC
siBRIX1-1
siBRIX1-2
siNC
siBRIX1-1
siBRIX1-2
E
DAPI
BRIX1
NPM1
MERGE
siNC
siBRIX1
F
CAL-51
siNC
siBRIX1-1
siBRIX1-2
CAL-51
***
***
Apoptotic rate (%)
siNC
siBRIX1-1
siBRIXI-2
G
MCF-7
siNC
siBRIX1-1
siBRIX1-2
MCF-7
***
***
Apoptotic rate (%)
siNC
siBRIX1-1
siBRIXI-2

Figure S3

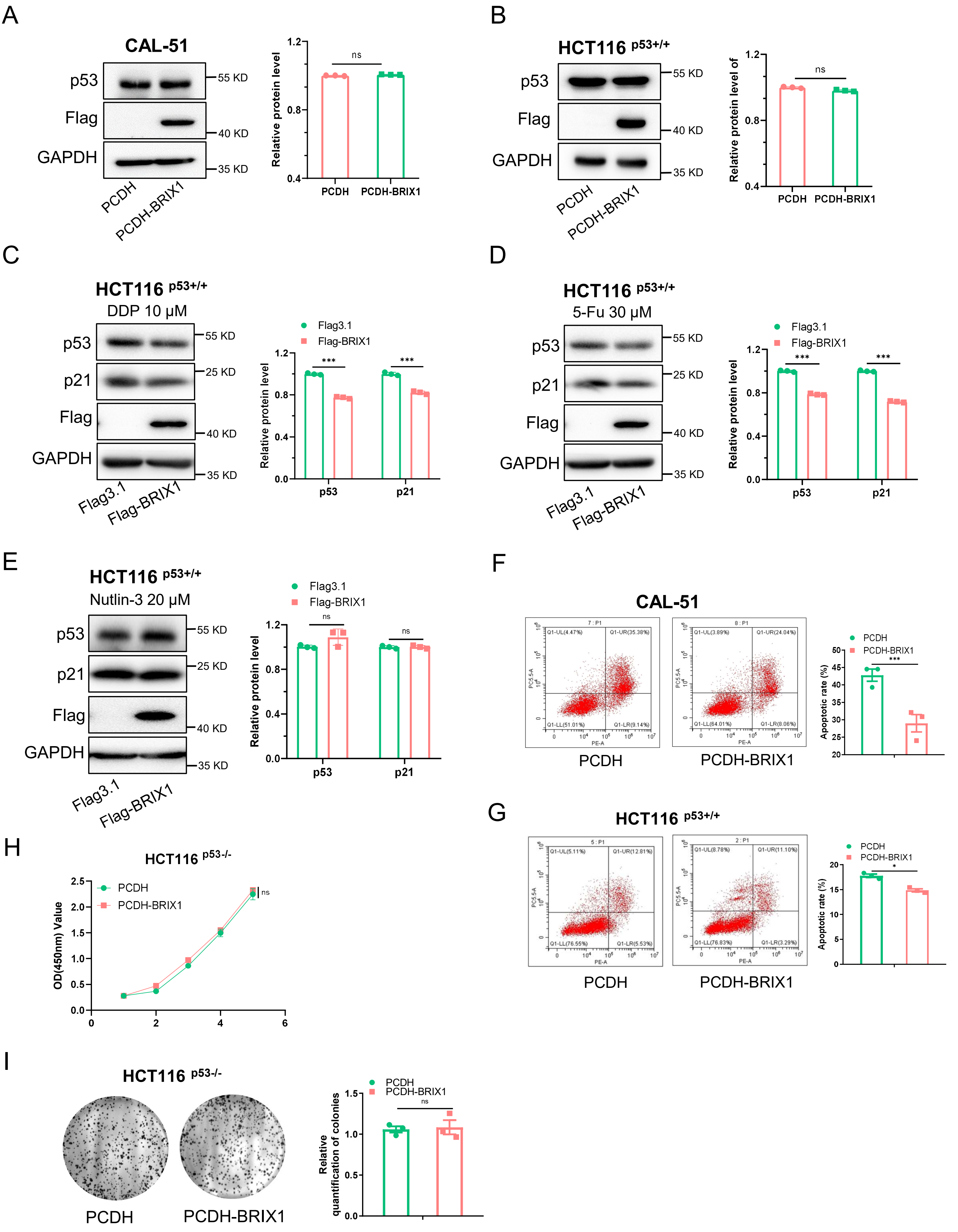

A
CAL-51
p53
Flag
GAPDH
55 KD
40 KD
35 KD
PCDH
PCDH-BRIX1
Relative protein level
ns
B
HCT116 p53+/+
Relative protein level of
C
DDP 10 μM
p21
25 KD
Flag3.1
Flag-BRIX1
***
D
5-Fu 30 μM
E
Nutlin-3 20 μM
F
Apoptotic rate (%)
G
*
H
HCT116 p53-/-
OD(450nm) Value
I
Relative quantification of colonies

Figure S4

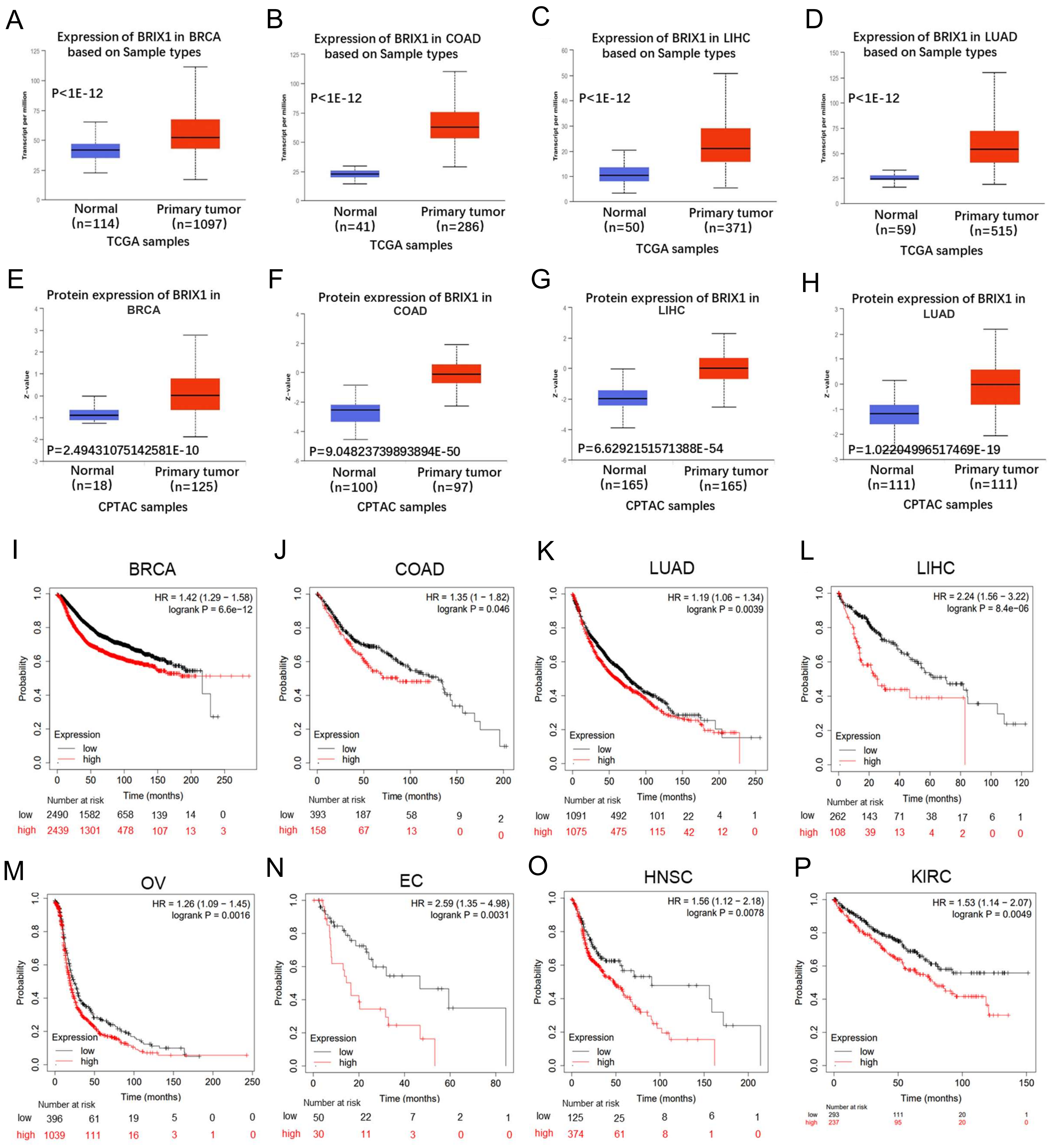
A
Expression of BRIX1 in BRCA based on Sample types
P<1E-12
Transcript per million
Normal (n=114)
Primary tumor (n=1097)
TCGA samples
B
Expression of BRIX1 in COAD based on Sample types
P<1E-12
Normal (n=41)
Primary tumor (n=286)
TCGA samples
C
Expression of BRIX1 in LIHC based on Sample types
P<1E-12
Normal (n=50)
Primary tumor (n=371)
TCGA samples
D
Expression of BRIX1 in LUAD based on Sample types
P<1E-12
Normal (n=59)
Primary tumor (n=515)
TCGA samples
E
Protein expression of BRIX1 in BRCA
Z-value
P=2.49431075142581E-10
Normal (n=18)
Primary tumor (n=125)
CPTAC samples
F
Protein expression of BRIX1 in COAD
P=9.04823739893894E-50
Normal (n=100)
Primary tumor (n=97)
CPTAC samples
G
Protein expression of BRIX1 in LIHC
P=6.6292151571388E-54
Normal (n=165)
Primary tumor (n=165)
CPTAC samples
H
Protein expression of BRIX1 in LUAD
P=1.02204996517469E-19
Normal (n=111)
Primary tumor (n=111)
CPTAC samples
I
BRCA
HR = 1.42 (1.29 − 1.58)
logrank P = 6.6e−12
Probability
Expression
low
high
Time (months)
Number at risk
low 2490 1582 658 139 14 0
high 2439 1301 478 107 13 3
J
COAD
HR = 1.35 (1 − 1.82)
logrank P = 0.046
Number at risk
low 393 187 58 9 2
high 158 67 13 0 0
K
LUAD
HR = 1.19 (1.06 − 1.34)
logrank P = 0.0039
Number at risk
low 1091 492 101 22 4 1
high 1075 475 115 42 12 0
L
LIHC
HR = 2.24 (1.56 − 3.22)
logrank P = 8.4e−06
Number at risk
low 262 143 71 38 17 6 1
high 108 39 13 4 2 0 0
M
OV
HR = 1.26 (1.09 − 1.45)
logrank P = 0.0016
Number at risk
low 396 61 19 5 0 0
high 1039 111 16 3 1 0
N
EC
HR = 2.59 (1.35 − 4.98)
logrank P = 0.0031
Number at risk
low 50 22 7 2 1
high 30 11 3 0 0
O
HNSC
HR = 1.56 (1.12 − 2.18)
logrank P = 0.0078
Number at risk
low 125 25 8 6 1
high 374 61 8 1 0
P
KIRC
HR = 1.53 (1.14 − 2.07)
logrank P = 0.0049
Number at risk
low 293 111 20 1
high 237 95 20 0

Figure S5

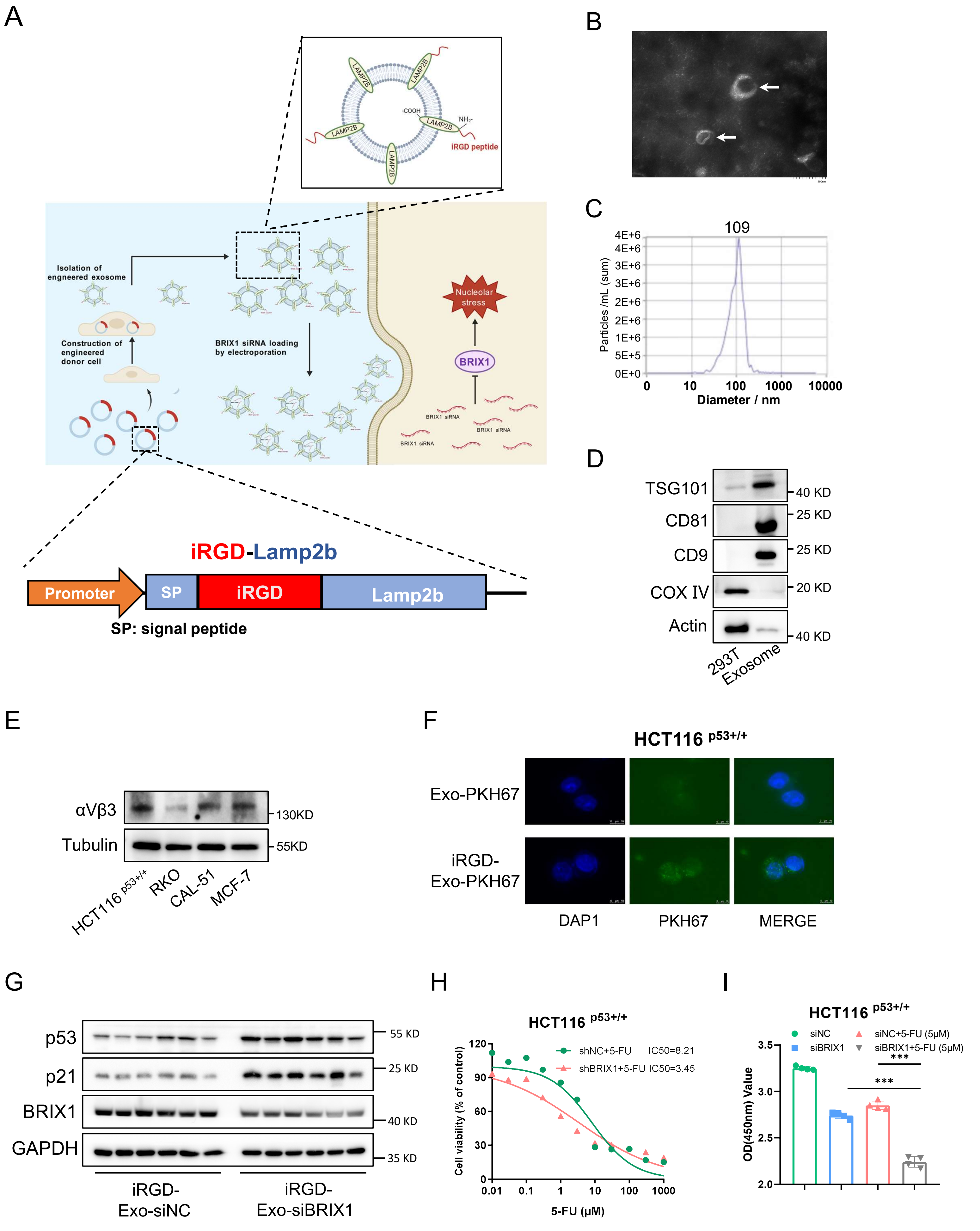
A
B
C
D
E
F
G
H
I
LAMP2B
-COOH
NH2-
iRGD peptide
Isolation of engneered exosome
Construction of engineered donor cell
BRIX1 siRNA loading by electroporation
Nucleolar stress
BRIX1
BRIX1 siRNA
iRGD-Lamp2b
Promoter
SP
iRGD
Lamp2b
SP: signal peptide
109
Particles /mL (sum)
Diameter / nm
TSG101
CD81
CD9
COX IV
Actin
40 KD
25 KD
25 KD
20 KD
40 KD
293T
Exosome
αVβ3
Tubulin
130KD
55KD
HCT116 p53+/+
RKO
CAL-51
MCF-7
HCT116 p53+/+
Exo-PKH67
iRGD-Exo-PKH67
DAP1
PKH67
MERGE
p53
p21
BRIX1
GAPDH
55 KD
25 KD
40 KD
35 KD
iRGD-Exo-siNC
iRGD-Exo-siBRIX1
HCT116 p53+/+
shNC+5-FU IC50=8.21
shBRIX1+5-FU IC50=3.45
Cell viability (% of control)
5-FU (μM)
HCT116 p53+/+
siNC
siBRIX1
siNC+5-FU (5μM)
siBRIX1+5-FU (5μM)
OD(450nm) Value
***
***

Supplementary Tables

Table S1: Relationship between BRIX1 expression and clinicopathologic factors of patients with breast cancer.

Variables	High（n=15）	%	Low（n=76）	%	p-value
	**BRIX1 Expression Level**				
Age（years）					
≥50	12	21.1	45	78.9	0.128
<50	3	8.8	31	91.2	
Tumor size					
⩾2cm	10	14.9	57	85.1	0.503
<2cm	5	20.8	19	79.2	
Lymph node					
N1-3	9	20	36	80	0.371
N0	6	13	40	87	

The comparison was determined by the Chi-square test or Fisher's exact test.

Table S2: Multivariate cox regression analysis of OS in 91 BRCA.

Variables	Univariate analysis		Multivariate analysis (forward stepwise)	
	HR (95%CI)	p-value	HR (95%CI)	p-value
BRIX1 (High vs. Low)	3.219 (1.320-7.848)	**0.01**	2.913 (1.158-7.329)	**0.023**
Age(years) (≥50 vs. <50)	2.998 (1.123-8.007)	**0.028**	3.060 (1.133-8.267)	**0.027**
Tumor size (≥2cm vs. <2cm)	0.894 (0.372-2.152)	0.803	-	-
Lymph node (N1-3 vs. N0)	3.128 (1.331-7.348)	**0.009**	3.331 (1.405-7.898)	**0.006**

Factors predicting OS were analyzed by univariate Cox's proportional hazard regression models. OS: overall survival; BRCA: breast cancer; HR: hazard ratio; CI: confidence intervals.

Table S3: Relationship between BRIX1 expression and clinicopathologic factors of patients with colorectal cancer.

Variables	BRIX1 Expression Level				p-value
	High（n=34）	%	Low（n=28）	%	
Age（years）					
<60	16	50	16	50	0.429
≥60	18	60	12	40	
Gender					
male	18	50	18	50	0.368
female	16	61.5	10	38.4	
Location					
colon	23	60.5	15	39.5	0.257
rectum	11	45.8	13	54.2	
Differentiation					
moderate or high	28	58.3	20	41.7	0.306
low	6	42.9	8	57.1	
TNM stage					
I+II	12	40	18	60	**0.023**
III+IV	22	68.7	10	31.3	

The comparison was determined by the Chi-square test or Fisher's exact test.

Table S4: Multivariate cox regression analysis of OS in 62 CRC.

Variables	Univariate analysis		Multivariate analysis (forward stepwise)	
	HR (95%CI)	p-value	HR (95%CI)	p-value
BRIX1 (high vs. low)	4.435 (1.489-13.210)	**0.007**	3.097 (1.012-9.474)	**0.048**
Age(years) (<60 vs. ≥60)	1.248 (0.526-2.963)	0.615	-	-
Gender (male vs. female)	1.266 (0.525-3.055)	0.600	-	-
Differentiation (moderate or high vs. low)	0.720 (0.279-1.860)	0.498	-	-
Location (colon vs. rectum)	1.241 (0.501-3.076)	0.641	-	-
TNM stage (I + II vs. III+IV)	0.193 (0.065-0.576)	**0.003**	0.260 (0.085-0.796)	**0.018**

Factors predicting OS were analyzed by univariate Cox's proportional hazard regression models. The factors with $P < 0.05$ in univariate Cox's regression were further analyzed in forward stepwise multivariate Cox's regression. OS: overall survival; HR: hazard ratio; CI: confidence interval.

Table S5. A list of primers for plasmid construction

Flag PCDNA3.1-EcoRI-BRIX1-F	5’-CGGAATTCGAATGGCGGCAACCAA-3’
Flag PCDNA3.1-XhoI-BRIX1-R	5’-CCGCTCGAGTTATTTTGTTTTCCCACTGT-3’
3Xflag-PCDH-EcoRI-BRIX1-F	5’-CGGAATTCATGGCGGCAACCAAGAGGAA-3’
3Xflag-PCDH-XhoI-BRIX1-R	5’-CCGCTCGAGTTATTTTGTTTTCCCACTGTCCATCCTCTG-3’
Myc PCDNA3.1-XbaI-BOP1-F	5’-GCTCTAGAATGGCGGGTTCGCGGGGTG-3’
Myc PCDNA3.1- EcoRI-BOP1-R	5’-CGGAATTCTGGTGAAGAGGCGGACAGTCCCGTCT-3’
Myc PCDNA3.1-PES1-F	5’-GCTCTAGAATGGGAGGCCTTGAGAAGAAGAAGT-3’
Myc PCDNA3.1-PES1-R	5’-CGGAATTCTCTCCGGCCTTGCCTTCTT-3’
Myc PCDNA3.1-WDR12-F	5’-GCTCTAGAATGGCTCAGCTCCAAACACG-3’
Myc PCDNA3.1-WDR12-R	5’-CGGAATTCTTGCCCCAACATGGGAAGT-3’
HA-PCMV-PES1-EcoRI-F	5’-CGGAATTCTCATGGGAGGCCTTGAGAAGAAGAAG-3’
HA-PCMV -PES1-KpnI-R	5’-CGGGTACCTCACTCCGGCCTTGCCTTC-3’

Table S6. A list of primers for RT-qPCR

GAPDH-F	5’-GGAGCGAGATCCCTCCAAAAT-3’
GAPDH-R	5’-GGCTGTTGTCATACTTCTCATGG-3’
p21-F	5’-CTGGACTGTTTTCTCTCGGCTC-3’
p21-R	5’-TGTATATTCAGCATTGTGGGAGGA-3’
BTG2-F	5’-ACGGGAAGGGAACCGACAT-3’
BTG2-R	5’-CAGTGGTGTTTGTAGTGCTCTG-3’
MDM2-F	5’-GAATCATCGGACTCAGGTACATC-3’
MDM2-R	5’-TCTGTCTCACTAATTGCTCTCCT-3’
BRIX1-F	5’-CGGAATTCGAATGGCGGCAACCAA-3’
BRIX1-R	5’-CCGCTCGAGTTATTTGTTTTCCCACTGT-3’
P53-F	5’-CCCAAGCAATGGATGATTTGA-3
P53-R	5’-GGCATTCTGGGAGCTTCATCT-3
28S-F	5’-AGAGGTCTTGGGGCCGAAACGATCTCAACC-3’
28S-R	5’-CTGATGAGCGTCGGCATCGGGCGCCTTAAC-3’
5S-F	5’-GGCCATACCACCCTGAACGC-3’
5S-F	5’-CAGCACCCGGTATTCCCAGG-3’
DHX35-F	5’-GGATGTGGGAAGAGCACACA-3’
DHX35-R	5’-GGATGTGGGAAGAGCACAC-3’
LSM6-F	5’-ATTGGCTGAGGCAGAAAAGTG-3’
LSM6-R	5’-ACAACTGGTCGTCCGATGATT-3’
PPAN-F	5’-TCCATAGCAACCGCTCCATC-3’
PPAN-R	5’-GACCTCCCTGACTGTCCCAT-3’
EXOSC6-F	5’-ACTCCACTAGGAATGCCGGA-3’
EXOSC6-R	5’-GGCTCAGAAAGACGTCGTGA-3’
EXOSC7-F	5’-GTCCAACACTAGTGGGTCCG-3’
EXOSC7-R	5’-AATTCAGGGGTAGCACTGGC-3’

RT-qPCR: real-time quantitative reverse transcription

Table S7. A list of siRNA sequences

siNC	5'- UUCUCCGAACGUGUCACGU-3'
siBRIX1-1	5'- CGUGUUUACUUUCACCAUU-3'
siBRIX1-2	5'- GCAUCGGCGUGUCAUAAGA-3'
siDHX35	5'- GGAAGAGUGGUAGGAGUGA-3'
siEXOSC6	5'-GAAUUGUUUAUCAGGAAUA-3'
siEXOSC7	5'-UAAAAGCAGUGUCGACUUA-3'
siLSM6	5'-GGAAACAAUGUGUUGUACA-3'
siPPAN	5'-GCGGUGGGAAAUGGAUCGA-3'
siRPL5	5'-GGAGGAGAUGUAUAAGAAA-3'
siRPL11	5'-GGAACUUCGCAUCCGCAAA-3'

Table S8. A list of primers for shRNA construction

shBRIX1-F	5'-CCGGGTGGCTTTCAAATTCACCTCACTCGAGTGAGGTGAATTTGAAAGCCACTTTTTG-3'
shBRIX1-R	5'-AATTCAAAAAGTGGCTTTCAAATTCACCTCACTCGAGTGAGGTGAATTTGAAAGCCAC-3'
